# Multiple Regulation of Rad51-Mediated Homologous Recombination by Fission Yeast Fbh1

**DOI:** 10.1371/journal.pgen.1004542

**Published:** 2014-08-28

**Authors:** Yasuhiro Tsutsui, Yumiko Kurokawa, Kentaro Ito, Md. Shahjahan P. Siddique, Yumiko Kawano, Fumiaki Yamao, Hiroshi Iwasaki

**Affiliations:** 1 Department of Biological Sciences, School and Graduate School of Bioscience and Biotechnology, Tokyo Institute of Technology, Meguro-ku, Tokyo, Japan; 2 Education Academy of Computational Life Science, Tokyo Institute of Technology, Meguro-ku, Tokyo, Japan; 3 International Institute for Advanced Studies, Kizugawa, Kyoto, Japan; Columbia University, United States of America

## Abstract

Fbh1, an F-box helicase related to bacterial UvrD, has been proposed to modulate homologous recombination in fission yeast. We provide several lines of evidence for such modulation. Fbh1, but not the related helicases Srs2 and Rqh1, suppressed the formation of crossover recombinants from single HO-induced DNA double-strand breaks. Purified Fbh1 in complex with Skp1 (Fbh1-Skp1 complex) inhibited Rad51-driven DNA strand exchange by disrupting Rad51 nucleoprotein filaments in an ATP-dependent manner; this disruption was alleviated by the Swi5-Sfr1 complex, an auxiliary activator of Rad51. In addition, the reconstituted SCF^Fbh1^ complex, composed of purified Fbh1-Skp1 and Pcu1-Rbx1, displayed ubiquitin-ligase E3 activity toward Rad51. Furthermore, Fbh1 reduced the protein level of Rad51 in stationary phase in an F-box-dependent, but not in a helicase domain-independent manner. These results suggest that Fbh1 negatively regulates Rad51-mediated homologous recombination via its two putative, unrelated activities, namely DNA unwinding/translocation and ubiquitin ligation. In addition to its anti-recombinase activity, we tentatively suggest that Fbh1 might also have a pro-recombination role *in vivo*, because the Fbh1-Skp1 complex stimulated Rad51-mediated strand exchange *in vitro* after strand exchange had been initiated.

## Author Summary

Homologous recombination is required for repairing DNA double-strand breaks (DSBs), which are induced by exogenous factors such as DNA damaging agents or by endogenous factors such as collapse of DNA replication fork in mitotic cells. If improperly processed, DSBs could lead to chromosome rearrangement, cell death, or tumorigenesis in mammals, and thus HR is strictly controlled at several steps, including Rad51 recombinase-driven DNA strand exchange reaction. Specifically, DNA helicases have been shown to be important for suppression of inappropriate recombination events. In this study, we analyzed one such DNA helicase, fission yeast Fbh1. We used an *in vivo* single-DSB repair assay to show that Fbh1 suppresses crossover formation between homologous chromosomes. Next, we obtained *in vitro* evidence that Fbh1 acts as an inhibitor of the strand-exchange reaction in the absence of Swi5-Sfr1, but stimulates the reaction after it starts. Furthermore, we found that SCF^Fbh1^ has ubiquitin-ligase activity toward Rad51 *in vitro* and that Fbh1 regulates the protein level of Rad51 in the stationary phase. These results suggest Fbh1 regulates Rad51-mediated homologous recombination by its seemingly-unrelated two activities, DNA helicase/translocase and ubiquitin ligase.

## Introduction

Homologous recombination (HR), a fundamental biological process in all organisms, involves the exchange of sequence between two homologous DNA molecules. In eukaryotes, HR plays an essential role in generating genetic diversity by exchanging paternal and maternal genomic information during meiosis, and also helps form the physical connections between homologous chromosomes that ensure faithful segregation in meiosis I. On the other hand, during mitotic cell growth, HR is involved in repair of DNA damage such as double-strand breaks (DSBs), or upon collapse of replication forks.

At a very early step in homologous recombination repair (HRR), the ends of a DSB are nucleolytically processed to produce recombinogenic DNA with 3′ single-strand overhangs. Eukaryotic recombinase Rad51 binds the resulting single-stranded DNA (ssDNA) region to form a nucleoprotein filament, and then catalyzes a strand-exchange reaction between the broken end and the homologous sequence, forming a D-loop structure. Auxiliary factors are required to form and activate the presynaptic filament of Rad51 recombinase. One such auxiliary factor is Rad52 (formerly called Rad22 in fission yeast), first identified in the budding yeast *Saccharomyces cerevisiae* (Sc) and humans; this protein is termed a recombination mediator. Rad52 facilitates Rad51-mediated strand exchange between replication protein A (RPA)–pre-coated ssDNA and homologous double-stranded DNA (dsDNA) [1]–[4]. Several other recombination mediators have been identified, including the Rad51 paralogs Rad55 and Rad57, which form a heterodimeric complex [5], and BRCA2, the product of human breast-cancer susceptibility gene 2 [6].

The Swi5-Sfr1 complex from the fission yeast *Schizosaccharomyces pombe* (Sp) represents a novel class of auxiliary factor. Both Swi5 and Sfr1 are widely conserved from yeast to humans [7]–[9]. The budding yeast Sae3-Mei5 complex, a homolog of Swi5-Sfr1 that is specifically expressed in early meiosis, has also been proposed to act as a mediator for the meiotic-specific recombinase Dmc1 [10], [11]. Genetic studies in fission yeast have shown that the Rad55-Rad57 and Swi5-Sfr1 complexes act in Rad51 sub-pathways that are independent of each other [7]. *In vitro*, the Swi5-Sfr1 complex stimulates Rad51 strand-exchange activity [12].

In addition to these auxiliary factors, Rad51-mediated HR is regulated by several DNA helicases such as Fbh1, Srs2, and the RecQ family helicases. Srs2 helicase, a superfamily-1 helicase closely related to bacterial UvrD, has been identified in budding yeast as a suppressor of the DNA damage sensitivity of a *rad6* mutants (suppressor of *rad six*) and a suppressor of hyper-recombination [13]–[15]. Several studies have suggested that Srs2 prevents HR during Rad6–Rad18 post-replication repair in budding yeast [reviewed in [16]]. In addition, Srs2 can disrupt Rad51 filaments and dissociate D-loop structures [17]–[19]. PARI has been identified as a human ortholog of yeast Srs2 [20].

The RecQ family helicases, which include ScSgs1, SpRqh1, HsBLM, HsWRN, and HsRecQL5, play a role in a late step of HR in which the double-Holliday junction is formed; this mature intermediate is dissolved by ScSgs1/HsBLM and topoisomerase III, yielding only non-crossover-type recombinants [21], [22]. Furthermore, BLM and RecQL5 dissociate human Rad51-ssDNA filaments in an ATPase-dependent manner [23], [24].

Fbh1 (F-box DNA helicase-1) was originally identified biochemically as a 3′ to 5′ DNA helicase in fission yeast by Seo and colleagues [25]. Subsequently, we independently cloned the *fbh1* ^+^ gene encoding this protein, which revealed it to be one of the *slr* genes (*slr5*), in which mutations are synthetically lethal with the *rad2Δ* mutation [26]. Whitby and colleagues also identified the same gene as a suppressor of a *rad52* mutation [27]. Mutations in either the DNA helicase or F-box motif of Fbh1 cause defects in HRR, suggesting that both the DNA helicase and ubiquitin-ligase activities of Fbh1 are involved in normal HRR [27], [28]. Further genetic studies have revealed that Fbh1 functions in a Rad51-dependent recombination pathway, and that *fbh1* mutations are synthetically lethal with *srs2Δ* or *rqh1Δ* mutations [26], [27]. Importantly, the lethality of the double mutants is suppressed by deletion of Rad51 or Rad57, implying that toxic HR intermediates produced in a Rad51- and Rad57-dependent manner are resolved by one of these three DNA helicases [26].

Interestingly, Fbh1 does not have a homolog in budding yeast, but homologs exist in multicellular eukaryotes. Genetic studies have implicated Fbh1 as a novel conserved regulator of recombination: Fbh1 suppresses sister chromatid exchange (SCE) and Rad51-mediated recombination at stalled replication forks during mitosis in several species [29]–[34].

The human homolog hFbh1 (originally named Fdh1) exhibits both DNA helicase activity and ubiquitin-ligase activity *in vitro* [35], [36]. However, it remains unclear how its DNA helicase activity is involved in HR and HRR. In addition, although the hSCF^Fbh1^ complex is required *in vivo* for degradation of the transcription factor Atf1 [37], a lack of Atf1 degradation cannot fully explain the phenotype of *fbh1* mutants carrying mutations in the F-box motif, strongly suggesting that Fbh1 ubiquitinates other physiologically important target protein(s).

In this study, we characterized SpFbh1 both *in vivo* and *in vitro* to reveal how this protein modulates HR though its helicase/translocase and ubiquitin-ligase activities. First, we showed that Fbh1 suppresses crossover (CO) formation, as revealed by analyses of repair of single DSBs. Second, we purified Fbh1, which could only be recovered as a heterodimer in complex with Skp1, and demonstrated that this complex plays two opposing roles in the DNA strand-exchange reaction: a negative role via disruption of Rad51 filament, and a positive role, via stimulation of the strand-exchange reaction. Finally, we reconstituted an *in vitro* ubiquitination reaction and showed that SCF^Fbh1^ E3 ligase efficiently promotes ubiquitination of Rad51.

## Results

### Fbh1 suppresses crossover during homologous recombination repair

We first investigated the functional differences among Fbh1, Srs2, and Rqh1 helicases using a site-specific DSBR assay system [38]. In this system, a single DSB is created by HO endonuclease at a 112 bp target site integrated into a nonessential minichromosome, Ch^16^-MG, which carries the *ade6-M216* allele. This single HO-induced DSB can be repaired by the non-homologous end-joining (NHEJ) pathway or by HRR involving Ch^16^-MG and Chromosome III. The various outcomes of induced DSBs can be inferred from the status of selectable markers: HRR yields a cell that is Ade^+^ G418^s^ or Ade^−^ G418^s^ (the latter of which can also result from minichromosome loss), whereas NHEJ and sister chromatid conversion (SCC) yields a cell that is Ade^+^ G418^r^. Pulsed-field gel electrophoresis (PFGE) analysis of independent Ade^+^ G418^s^ and Ade^−^ G418^s^ colonies from each assay can reveal whether HRR-mediated survivors arise by gene conversion (GC), crossover (CO), non-reciprocal exchange (BIR), or long-tract gene conversion (LTGC) (Figure S1) [8], [38]. Note that this assay cannot discriminate between Ade^+^ G418^r^ segregants arising from NHEJ and SCC [39].

We quantitated the outcomes of single-DSB induction by monitoring G418^r^ and Ade^+^ marker loss in a colony assay (Table S1). Based on the results, three categories of outcomes (NHEJ/SCC, HRR, and minichromosome loss) are displayed in Table 1. HRR, accounting for 72.9% of the outcomes in the wild-type strain, is a main repair pathway for single DSBs in this assay, as reported previously [8], [38]. HRR was reduced in all deletion mutants of individual helicases (*rqh1Δ*, *srs2Δ*, and *fbh1Δ*), indicating that these helicases are involved in DSB repair mediated by HRR: the *rqh1Δ* mutation caused the most drastic effect (12.9%), whereas *srs2Δ* and *fbh1Δ* reduced HRR more modestly (43.0% and 52.5%, respectively). By contrast, the levels of NHEJ/SCC in the three helicase mutants were significantly elevated, especially in the case of *rqh1Δ* (70.4% vs. 10.3% in the wild type). Previously, Hope *et al.* reported similar results: in the *rqh1Δ* mutant, most segregants were Ade^+^ G418^r^ (the products of NHEJ and SCC), whereas GC was drastically decreased. Given that HO endonuclease efficiently cuts Ch^16^-MG even in the *rqh1Δ* background, and that Ade^+^ G418^r^ segregants are independent of Ku, Hope *et al.* concluded that most Ch^16^-MG molecules cut by HO endonuclease are repaired by SCC in the *rqh1Δ* background [39]. In the *srs2Δ* and *fbh1Δ* mutants, the rates of NHEJ/SCC segregants were 42.7% and 43.4%, respectively.

**Table 1:** Summary of the outcomes of single-DSB induction (%).

Strain	NHEJ/SCC	HRR	Minichromosome loss
***wild-type***	10.3±2.0	72.9±7.3	13.8±4.5
***rad51Δ*** a	16.7±0.6	19.8±1.8	63.5±2.0
***swi5Δ*** a	33.1±2.6	36.4±2.3	30.5±2.0
***sfr1Δ*** a	25.5±3.4	43.0±2.4	31.5±2.6
***rad57Δ*** a	19.8±3.3	37.4±4.4	42.8±4.5
***rqh1Δ***	70.4±1.7	12.9±0.3	16.7±0.3
***srs2Δ***	42.7±2.3	43.0±2.1	12.8±1.0
***fbh1Δ***	43.4±3.3	52.5±0.7	2.4±0.7
***rad51Δ fbh1Δ***	17.5±2.0	5.2±0.0	77.3±0.0
***swi5Δ fbh1Δ***	32.2±7.6	62.7±1.0	3.9±0.1
***sfr1Δ fbh1Δ***	33.1±2.7	57.1±4.0	4.6±0.9
***rad57Δ fbh1Δ***	56.9±1.6	36.3±0.1	5.5±0.1

Colonies that arose after HO induction were classified into three categories: NHEJ, HRR, and minichromosome loss, based on phenotypic and PFGE analyses. The average frequencies (total segregants = 100%) and standard errors were determined from the data sets in Tables S1, S2, S3.

a Data are from Akamatsu et al. (2007) [8].

NHEJ, non-homologous end-joining; SCC, sister chromatid conversion; HRR, homologous recombination repair; PFGE, pulsed-field gel electrophoresis.

We then investigated the possibility that SCC is elevated in these mutants, as in the case of the *rqh1Δ* mutant [39], by measuring the efficiency of HO digestion (Figure S2). First, we determined the relative copy numbers of the *act1* and *ade6* genes (the *ade6*/*act1* ratio). Because the *ade6* gene is carried by both Chromosome III and minichromosome Ch16-MG, the *ade6*/*act1* ratio before HO induction should be ∼2; after Ch16-MG is lost, the ratio should be ∼1. As shown in Figure S2B, the *ade6*/*act1* ratios in the three helicase mutants were ∼2, similar to the ratio in the wild-type strain, indicating that very little reduction occurred after HO induction; thus the minichromosome was retained in most mutant cells, even after HO induction. Next, we determined the *HOcs*/*act1* ratios in the helicase mutants (Figure S2C). We determined that the digestion efficiency was 71.8% in wild type, 55.0% in *fbh1Δ*, 58.8% in *srs2Δ*, and 56.4% in *rqh1Δ*, as determined by the formula %efficiency = (1-[*HOcs/act1* with HO induction]/[*HOcs/act1* without HO induction])×100 (see [39]), indicating that DSBs were formed very efficiently upon HO induction. Therefore, we concluded that the elevated rates of SCC/NHEJ in the three helicase mutants were not primarily due to increases in the undigested population. Sequence analysis of the *HOcs* region in 48 independent Ade^+^Kan^r^ clones revealed that fewer than 4.2% of clones in each strain were repaired by NHEJ (Figure S2D). Therefore, these colonies most likely represent products of SCC rather than NHEJ. The elevation of SCC in the *fbh1Δ* mutant was consistent with previous reports [32], [40]. On the other hand, minichromosome loss was drastically reduced in the *fbh1Δ* single mutant (2.4% in *fbh1Δ* vs. 13.8% in the wild type), but drastically increased by the *rad51Δ* mutation (77.3%) (Table 1), suggesting that Rad51 plays an important role in maintenance of DSB-introduced minichromosomes and that Fbh1 suppresses Rad51 function(s).

Evaluation of the outcomes of HRR by PFGE revealed that GC was reduced to about 50% of the wild-type level in the *fbh1Δ* mutant (28.7% in *fbh1Δ* vs. 56.6% in the wild type), whereas CO was markedly higher (21.8% in *fbh1Δ* vs. 5.1% in the wild type) (Table 2). To investigate the genetic interactions between Fbh1 and other HRR factors, we introduced *rad51Δ*, *swi5Δ*, and *rad57Δ* mutations into the *fbh1Δ* mutant background. The *rad51Δ fbh1Δ* mutant completely abolished CO, indicating that the CO observed in the *fbh1Δ* mutant was dependent on *rad51^+^*. In fission yeast, the Rad51 pathway diverges into two genetically independent sub-pathways, Rad55-Rad57 and Swi5-Sfr1 [7]. The CO percentage in the *rad57Δ fbh1Δ* mutant (7.8%) was only marginally higher than that in the wild-type strain, and was significantly lower than the corresponding rate in the *swi5Δ fbh1Δ* mutant (14.2%) and the *sfr1Δ fbh1Δ* mutant (15.7%), suggesting that the elevated CO rate in the *fbh1Δ* mutant depends primarily on the Rad55-Rad57 sub-pathway.

**Table 2:** Outcomes of HRR among survivors (% of total).

Strain	GC	CO	LTGC	BIR type 1	BIR type 2	two-marker GC w/CO	Unknown
***wild-type***	56.6±4.4	5.1±3.3	9.5±4.9	0.7±1.3	0.5±0.5	0.5±0.5	3.4±4.6
***rad51Δ*** a	5.0±0.6	ND^b^	13.2±1.1	ND^b^	0.8±0.7	0.8±0.7	ND^b^
***swi5Δ*** a	21.8±1.4	1.9±0.6	12.1±1.6	0.2±0.2	0.4±0.4	ND^b^	ND^b^
***sfr1Δ*** a	26.6±1.7	1.4±0.5	14.2±1.8	ND^b^	0.8±0.7	ND^b^	ND^b^
***rad57Δ*** a	9.6±0.8	ND^b^	27.0±3.9	ND^b^	ND^b^	0.6±0.5	0.3±0.1
***rqh1Δ***	5.6±1.0	6.0±0.7	0.3±0.3	0.4±0.4	0.6±0.0	ND^a^	0.6±0.0
***srs2Δ***	33.5±5.4	7.5±5.4	1.6±1.5	ND^b^	0.2±0.3	0.2±0.3	1.6±1.4
***fbh1Δ***	28.7±2.0	21.8±2.6	1.1±1.0	ND^b^	ND^b^	0.9±0.3	1.6±0.1
***rad51Δ fbh1Δ***	5.1±0.1	ND^b^	ND^b^	0.1±0.0	ND^b^	ND^b^	ND^b^
***swi5Δ fbh1Δ***	45.2±1.0	14.2±1.7	2.1±0.1	1.0±1.2	0.1±0.1	0.4±0.0	1.2±1.0
***sfr1Δ fbh1Δ***	39.4±7.8	15.7±9.9	0.8±0.2	2.0±1.7	0.3±0.3	0.4±0.4	5.4±2.7
***rad57Δ fbh1Δ***	25.9±2.2	7.8±0.4	1.9±0.1	0.8±0.7	ND^b^	0.3±0.0	1.2±1.2

The average frequencies of colonies produced by HRR (total HO-induced segregants = 100%) and standard errors were determined from the data sets in Tables S1, S2, S3.

a Data are from Akamatsu et al. (2007) [8].

b No colonies were obtained.

BIR, break induced replication; CO, crossover; GC, gene conversion; LTGC, long-tract gene conversion.

Although both budding yeast and fission yeast Srs2 suppress CO formation, as demonstrated by multiple assays [13], [22], [41], [42], the *srs2Δ* mutation resulted in only a slight increase in CO in our assay (7.5%). This finding indicates that Srs2 is less important than Fbh1 for suppression of CO production from DSBRs, at least in this site-specific DSBR assay. The *rqh1Δ* mutation also had a very slight effect on CO production (6.0%).

### Purification of Fbh1-Skp1 complex

To examine the function of Fbh1 biochemically, we purified recombinant proteins using a baculovirus system. Because we could not purify Fbh1 alone, we purified Fbh1 and Skp1 as a protein complex (Figure S3A). We also purified (in complex with Skp1) Fbh1^K301A^, an Fbh1 mutant containing a substitution in an amino acid in the Walker A box that is critically important for ATPase activity, as well as Skp1 alone. To assess whether the Fbh1-Skp1 complex is functional *in vitro*, we measured its ATPase activity in the presence or absence of DNA (Figure S3B). The Fbh1-Skp1 heterodimer exhibited a robust, ssDNA-dependent ATPase activity. Because neither Fbh1^K301A^-Skp1 nor Skp1 alone exhibited ATPase activity, we concluded that the observed ATPase activity was due to wild-type Fbh1 (Figure S3C). Next, we investigated the DNA-binding activity of Fbh1 by performing gel mobility-shift assays using ssDNA or double-stranded DNA (dsDNA); the results revealed that Fbh1 preferentially binds ssDNA (Figure S3D). Because Fbh1 has 3′ to 5′ DNA helicase activity [25], we subjected the purified Fbh1-Skp1 complex to a DNA helicase assay. Fbh1 unwound heteroduplex DNA with a 3′ overhang, but not a duplex or a heteroduplex with a 5′ overhang. Thus, as expected, the Fbh1-Skp1 complex exhibited helicase activity with 3′ to 5′ directionality (Figure S3E).

### Fbh1 disrupts Rad51 filaments

**Figure 1:**
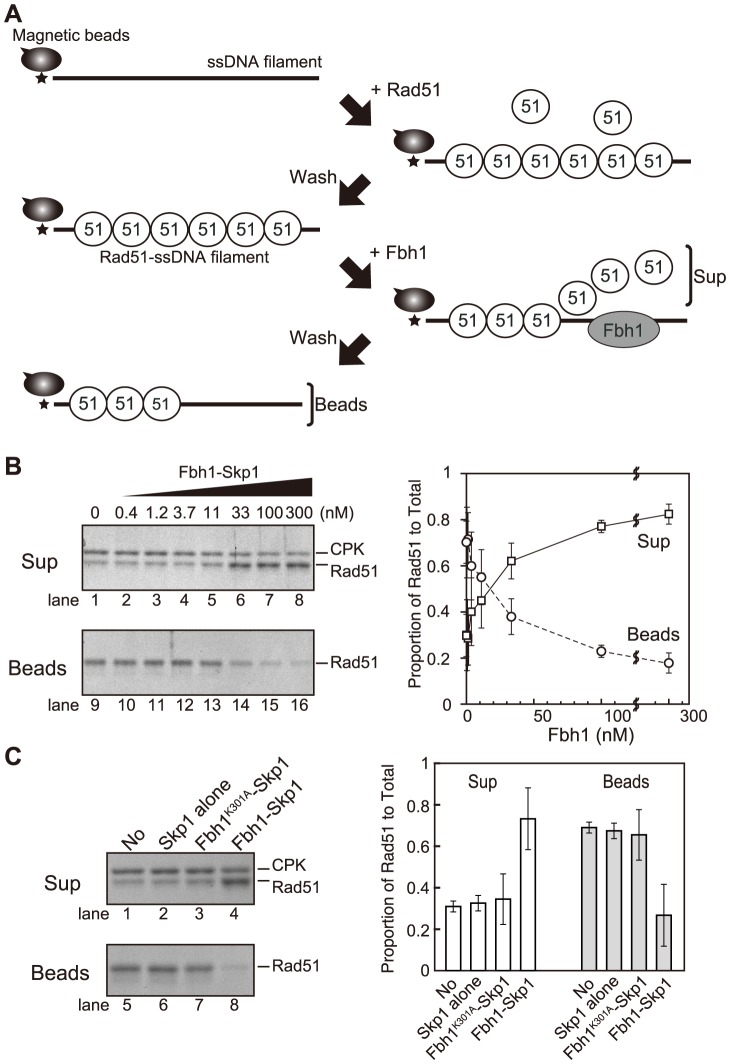
Disruption of Rad51-ssDNA filaments is dependent on Fbh1 helicase/translocase activity. (A) Schematic diagram of the ssDNA-bead assay. After preparation of ssDNA beads, Rad51 is incubated to form Rad51-ssDNA filaments. After washing, Rad51-ssDNA filament was challenged with the Fbh1-Skp1 complex. After incubation, the supernatant (unbound) and bead (bound) fractions were analyzed by SDS-PAGE and visualized by CBB-G250 staining. (B) The effect of Fbh1-Skp1 dose on stability of the Rad51-ssDNA filament. Left, gel image of the assay; right, quantification of Fbh1-Skp1 dose effects. Images were captured, and the intensities of Rad51 protein bands were quantified. Summary of quantification is presented in the graph at right. The SDS-PAGE gel in the upper image was 9% (19∶1 acrylamide∶bisacrylamide); this condition was used to achieve better separation of CPK and Rad51 bands. The gel in the lower image was 9% (29∶1 acrylamide∶bisacrylamide). Each mean value and standard error (SE) was obtained from three independent experiments. (C) Fbh1, but not Skp1, displaces Rad51 from ssDNA. The ssDNA-bead assay was carried out using wild-type Fbh1, the Walker A mutant of Fbh1 (Fbh1^K301A^), or Skp1 alone. Left, gel image of the assay. The two SDS-PAGE gels were 9% (19∶1 acrylamide∶bisacrylamide). Right, proportion of Rad51 recovered in the indicated fraction, relative to total amount of Rad51; each mean value and SE was obtained from three independent experiments.

Srs2 and Fbh1 are predicted to have similar biochemical activity, based on their structural relatedness to bacterial UvrD helicase. ScSrs2 disrupts ScRad51-ssDNA nucleoprotein filaments [17], [18]. Fbh1 has also been proposed to disrupt Rad51-ssDNA filaments based on its relationship to Srs2 and on the results of *in vivo* analyses in both fission yeast and humans [29], [33], [43]. Thus, we first investigated whether Fbh1 affects the stability of Rad51-ssDNA nucleoprotein filaments *in vitro* by preparing Rad51-ssDNA filaments using biotinylated ssDNA-coated beads (Figure 1A) and exposing them or not to Fbh1. In the absence of Fbh1-Skp1, Rad51 was found mostly in the bound fraction, indicating that it bound stably to the ssDNA beads (Figure 1B, lanes 1 and 9). However, in the presence of Fbh1-Skp1, Rad51 was found in the unbound fraction, indicating that Fbh1-Skp1 disrupted the Rad51-ssDNA filaments. The effect of Fbh1-Skp1 was dose-dependent (Figure 1B). The disruption of Rad51-ssDNA filaments by Fbh1-Skp1 was dependent on Fbh1 helicase/translocase activity, because Rad51 remained bound to the ssDNA beads in the presence of a Walker A mutant of Fbh1 (Fbh1^K301A^-Skp1) or Skp1 alone (Figure 1C). Thus, we conclude that Fbh1 can disrupt Rad51 filaments via its helicase/translocase activity.

### Swi5-Sfr1, an activator protein complex of Rad51, protects Rad51 filaments against Fbh1-mediated disassembly

**Figure 2:**
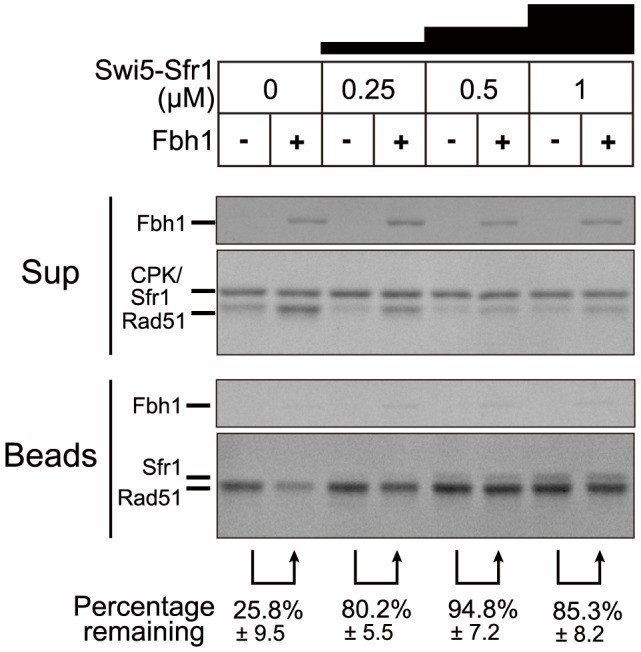
The Swi5-Sfr1 activator complex blocks the Fbh1-mediated Rad51 dissociation from ssDNA. The ssDNA-bead assay was carried out as described in Figure 1. Intensity of Rad51 was quantified, and percentage reduction at each concentration of Swi5-Sfr1 was calculated as [(Rad51 amount on Beads in the presence of Fbh1)/(Rad51 amount on Beads in the absence of Fbh1)×100]. Upper panel shows the amount of Fbh1 in each lane. Each value and SE was obtained from three independent experiments.

Because the Swi5-Sfr1 complex activates Rad51 filaments [44], we considered the possibility that the Swi5-Sfr1 complex confers resistance to Rad51 displacement by Fbh1. To address this, we investigated whether Fbh1 disrupts Rad51-ssDNA nucleoprotein filament in the presence of the Swi5-Sfr1 complex. Indeed, Fbh1 disrupted Rad51-ssDNA nucleoprotein filaments in the absence, but not the presence, of Swi5-Sfr1 (Figure 2). In this assay, we used 5 µM Rad51, the same concentration used in the DNA strand-exchange assay that we established previously [12]. The DNA strand-exchange assay revealed that 0.5 µM Swi5-Sfr1 is sufficient for stimulation. Consistent with this, a similar concentration of Swi5-Sfr1 (0.25–1 µM) confers resistance to Rad51 displacement by Fbh1, probably via the activation of Rad51-ssDNA nucleoprotein filaments. During the preparation of this manuscript, another group reported that hFbh1 forms stable complexes with active hRad51 nucleoprotein filaments in the presence of Ca^2+^ [45]. To determine whether a similar interaction occurs between the fission yeast homologs of these proteins, we investigated whether Fbh1 binds active Rad51 filaments in the presence of the Swi5-Sfr1 complex (Figure 2). No significant increase in the amount of bead-bound Fbh1 was observed, suggesting either that SpFbh1 does not form a stable complex with SpRad51 nucleoprotein filaments, or that the state of SpRad51 filaments in the presence of Swi5-Sfr1 complex is different from that of hRad51 filaments activated by Ca^2+^.

### Impacts of Fbh1 on the Rad51-Swi5-Sfr1-driven strand-exchange reaction

**Figure 3:**
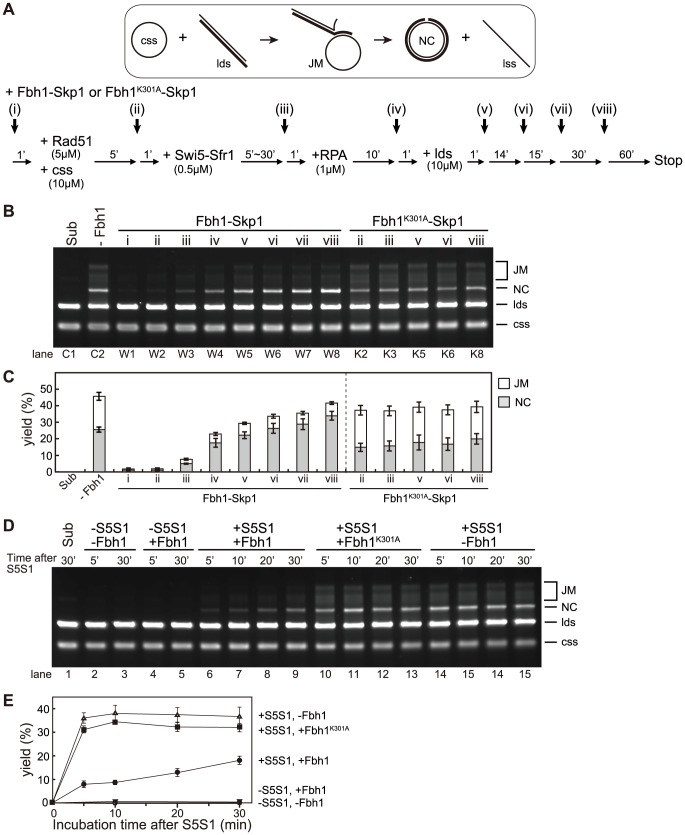
Fbh1 inhibits the strand-exchange reaction at early steps, but stimulates it at the late steps. (A) Schematic of the three-strand exchange reactions. Fbh1 (90 nM) was added at the indicated step, (i)–(viii). (B) Gel image of three-strand exchange reactions. (C) Quantitation of results in panel B. Each mean value and SE was obtained from three independent experiments. (D) Three-strand exchange reactions were carried out with the indicated incubation times after addition of Swi5-Sfr1 (5–30 min). (E) Quantitation of results in panel D. Each mean value and SE was obtained from three independent experiments.

Next, we investigated whether Fbh1 inhibits the three-strand DNA exchange reaction mediated by Rad51, using a previously developed assay that allowed us to monitor homology search and strand exchange between ssDNA and dsDNA catalyzed by Rad51 recombinase [12]. As expected, both the reaction intermediates (joint molecules, JMs), and the final products (P, also referred to as the nicked circle, NC, and single-stranded linear DNA) were almost completely abolished if Fbh1-Skp1 was added at early steps (i) or (ii) when the Swi5-Sfr1 complex was absent (Figure 3B, lanes W1 and W2). Fbh1^K301A^-Skp1 had very little effect (Figure 3B, lane K2); therefore, inhibition of the three-strand DNA exchange reaction was dependent on the helicase/translocase activity of Fbh1. By contrast, this inhibition was largely alleviated if Fbh1-Skp1 was added after step (iii) (Figure 3B, lanes W3 and W4). These results are consistent with the idea that Fbh1 disrupts Rad51-ssDNA filaments in the absence of the Swi5-Sfr1 complex, although full restoration of the strand-exchange reaction was not achieved by the addition of Swi5-Sfr1. One possible explanation for this finding is that short incubation time limited the activation of Rad51 filament by Swi5-Sfr1. To test this idea, we allowed Swi5-Sfr1 to activate Rad51 filament for various incubation times (5–30 min), and then added Fbh1 at step (iii) (Figure 3D). As shown in Figure 3E, the yield of three-strand exchange increased upon longer incubation. We also evaluated the amounts of bead-bound or free Rad51 protein resulting from addition of Fbh1 at different times, using ssDNA beads (times [I] and [II] in Figure S4A). In this experiment, we used almost the same conditions as in the strand-exchange reaction, but incubated for 60 min before collecting the beads. Addition of Fbh1 after incubation with Swi5-Sfr1 (i.e., time [II]) slightly increased the amount of bead-bound Rad51 relative to addition of Fbh1 time [I]. Taken together, the result indicated that Swi5-Sfr1 confers resistance to the helicase/translocase activity of Fbh1 in the strand-exchange reaction.

Surprisingly, the level of NC increased when Fbh1-Skp1 was added at step (v) or later (Figure 3B, lanes W5–W8), whereas the level of JMs decreased. These results indicate that Fbh1-Skp1 promotes the strand-exchange reaction of already formed JMs. This promotion also requires the helicase/translocase activity of Fbh1, because Fbh1^K301A^-Skp1 was not effective in this respect (Figure 3B, lanes K5, K6, and K8). The stimulatory effect was not observed in the absence of Swi5-Sfr1 (Figure S5).

One possible explanation for the observed stimulation is that the 3′ end of the displaced strand accessible to Fbh1 may unwind the linear dsDNA via its 3′ to 5′ DNA helicase activity, allowing the NC to form by simple annealing. To address this possibility, we investigated whether Fbh1 stimulates the strand-exchange reaction mediated by RecA (Figure S6). However, Fbh1 inhibited the three-strand exchange rather than stimulating it (Figure S6B lanes 3–5), indicating that the formation of the accessible 3′ end of the displaced strand is not sufficient for stimulation.

Based on these results, we propose that Fbh1 plays both inhibitory and stimulatory roles in the Rad51-mediated strand-exchange reaction.

### Fbh1 ubiquitinates Rad51

A previous genetic study revealed that both the helicase and F-box motifs are required for full Fbh1 functionality [28], suggesting that Fbh1 has ubiquitin-ligase (E3) activity in addition to helicase/translocase activity. In general, F-box proteins form SCF E3 complexes including Cullin1, Skp1, and Rbx1 proteins [46]. The human ortholog (HsFbh1) forms the HsSCF^Fbh1^ complex, which has ubiquitin-ligase activity *in vitro*, although its target proteins remain unknown [35], [36]. Because the Fbh1-Skp1 complex physically and functionally interacts with Rad51 *in vitro* (Figures 1–3 and S7A), we suspected that Rad51 might be also a target of the E3 complex.

**Figure 4:**
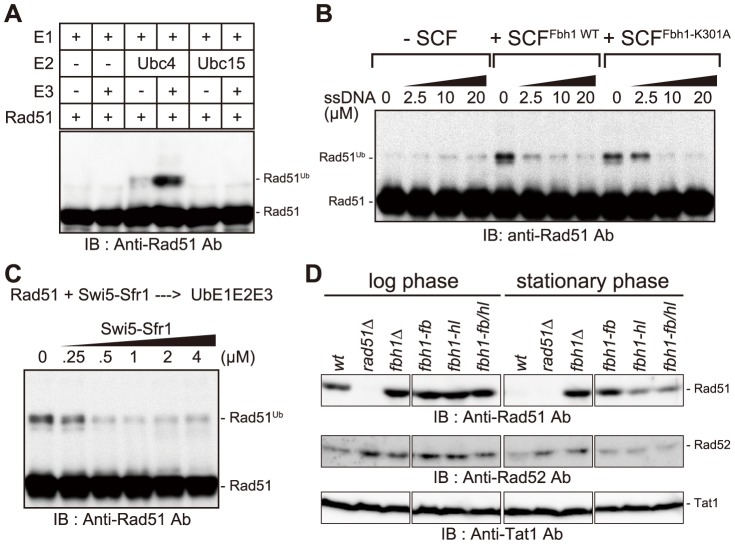
Rad51 is ubiquitinated *in vitro* in an SCF^Fbh1^-dependent manner. (A) Two kinds of E2 enzymes, Ubc4 and Ubc15, were used in the *in vitro* ubiquitination assays. After the reaction, the reaction mixture was analyzed by western blotting with anti-Rad51 antibody. Rad51 was ubiquitinated in a Ubc4- and SCF^Fbh1^-dependent manner. (B) The helicase activity of Rad51 is dispensable for its ubiquitination, and ssDNA inhibited Rad51 ubiquitination. Both SCF^Fbh1WT^ and SCF^Fbh1-K301A^ promoted Rad51 ubiquitination, but no increase in ubiquitination was observed in the absence of SCF. (C) *In vitro* ubiquitination assay containing Swi5-Sfr1. Swi5-Sfr1 inhibited Rad51 ubiquitination in a dose-dependent manner. (D) Comparison of Rad51 and Rad52 protein levels in log and stationary phases. Rad51 protein was degraded in stationary phase in wild-type cells, but this degradation was blocked by *fbh1* mutations; consequently, Rad51 protein levels were higher in *fbh1* mutants than in the wild-type strain.

To obtain the SpSCF^Fbh1^ complex, we separately purified Fbh1-Skp1 and Pcu1 (fission yeast Cullin1)-Rbx1 complexes, mixed them to form the SpSCF^Fbh1^ complex, and used these recombinant proteins in an *in vitro* ubiquitination assay. Several SCF complexes have ubiquitin E3 ligase activity that requires either Ubc4 or Cdc34 as the E2 enzyme [47]. In fission yeast, Ubc15 is a candidate functional homolog of Ccd34. Thus, we tested both Ubc4 and Ubc15 as candidate E2 enzymes in a ubiquitination assay with the SCF^Fbh1^ complex. As shown in Figure 4A, Rad51 was ubiquitinated in an Ubc4- and SCF^Fbh1^-dependent manner.

We also tested commercially available human E2 enzymes. Rad51 was ubiquitinated efficiently in reactions containing three UbcH5 variants, but was not ubiquitinated in reactions containing UbcH13/Cdc34 (Figures S7B and S7C). This result is consistent with the results obtained in the *S. pombe* ubiquitination assay, because SpUbc4 and SpUbc15 are considered to be functional counterparts for UbcH5 proteins and UbcH13/Cdc34, respectively (Figure S8). Therefore, we conclude that Ubc4 is the functionally cognate E2 for the SpSCF^Fbh1^ E3, at least for the *in vitro* ubiquitination of Rad51.

The SCF^Fbh1-K301A^ complex exhibited activity similar to that of SCF^Fbh1^ (Figure 4B), indicating that SCF^Fbh1^ E3 activity towards Rad51 is not dependent on its translocase activity. Therefore, we investigated whether Rad51 is ubiquitinated in the presence of ssDNA. However, the ubiquitination of Rad51 was inhibited by ssDNA, suggesting that Rad51-filament might not be the cognate substrate for the ubiquitination reaction (Figure 4B).

### Swi5-Sfr1 inhibits ubiquitination of Rad51

*In vitro* ubiquitination of Rad51 was inhibited by the Swi5-Sfr1 complex in a dose-dependent manner (Figure 4C). Because Fbh1 appears not to interact with Swi5-Sfr1 (Figure S7A), Swi5-Sfr1 might occlude the ubiquitination site and/or induce a structural change in Rad51 that prevents ubiquitination by SCF^Fbh1^. Taken together with the observation that Swi5-Sfr1 inhibited the disruption of Rad51 filaments by the helicase/translocase activity of Fbh1 (Figure 3), this finding indicates that Swi5-Sfr1 protects Rad51 against both of the inhibitory activities of Fbh1 described above.

### Fbh1 affects Rad51 stability in stationary phase

Based on our observation that the SCF^Fbh1^ complex ubiquitinates Rad51 *in vitro*, we next investigated whether Rad51 is ubiquitinated *in vivo*. However, we could not detect ubiquitinated Rad51 in cells treated with several DNA-damaging agents (Unpublished observation), possibly because ubiquitination of Rad51 is transient and/or inefficient. In the course of this analysis, we noticed that, in stationary phase, the Rad51 protein level decreased drastically in wild-type cells (Figure 4D), but increased in several *fbh1* mutants. In addition, we found that the Rad51 stability in an F-box mutant, *fbh1-fb*, was similar to that in the *fbh1Δ* mutant, whereas the Rad51 stabilities in a helicase-dead mutant, *fbh1-hl* (*fbh1^K301A^*), and a double mutant, *fbh1-fb/hl*, were lower. These results suggest that Fbh1 regulates the level of Rad51 protein in stationary phase, which is dependent on its F-box motif. Conversely, Rad52 is more stable than Rad51, even during stationary phase, in both the wild-type and *fbh1* mutants (Figure 4D).

## Discussion

In this study, we characterized the *in vivo* and *in vitro* roles of fission yeast Fbh1. Comparisons of the activities of *rqh1Δ*, *srs2Δ*, and *fbh1Δ* mutants in a site-specific DSBR assay revealed that Fbh1, but not Srs2, is important for the suppression of CO formation. Biochemical analysis showed that Fbh1 disrupts Rad51-ssDNA filaments in the absence, but not in the presence, of the Swi5-Sfr1 complex. Consistent with this, Fbh1 negatively regulated the Rad51-catalyzed strand exchange before the start of the reaction, especially when the Rad51 filament was not activated by the Swi5-Sfr1 complex. However, once the reaction started, Fbh1 stimulated the strand-exchange reaction. Furthermore, the SCF^Fbh1^ complex ubiquitinated Rad51 *in vitro*. Based on these observations, we suggest that Fbh1 performs an anti-recombinase role via its helicase/translocase activity and ubiquitin ligase activity. Furthermore, we suspect that Fbh1 might also promote the strand exchange reaction under similar conditions.

### Fbh1 suppresses CO formation during interchromosomal recombination

Using the same single-DSBR analysis employed in this study, we previously showed that Rad55-Rad57 is essential for CO formation in the *fbh1* ^+^ background, whereas the Swi5-Sfr1 pathway is not [8]. In this study, we demonstrated that Fbh1 is important for the suppression of CO (Table 2). Elevated CO formation in the *fbh1Δ* mutant largely depended on the Rad55-Rad57 sub-pathway, rather than on the Swi5-Sfr1 sub-pathway (Table 2). Moreover, Whitby and colleagues showed that deletion of *fbh1* has no effect on spontaneous intrachromosomal recombination [27]. Taken together, these findings suggest that Fbh1 negatively regulates the Rad55-Rad57 sub-pathway of Rad51-dependent HR to suppress CO formation during interchromosomal recombination.

Fission yeast has three functionally interdependent DNA helicases, Fbh1, Srs2, and the RecQ helicase Rqh1. By contrast, budding yeast has Srs2 and the RecQ helicase Sgs1, but no apparent Fbh1 homolog; however, some functions of Fbh1 may be served by Srs2, which suppresses CO in budding yeast [13], [22], [42]. Metazoans such as human and mouse have Fbh1, the Srs2 homolog PARI [20], and several RecQ homologs, including WRN, BLM, and RecQ5. *HsFBH1* partially complements the phenotype of the budding yeast *srs2Δ* mutant [48], suggesting that Fbh1 and Srs2 are functionally redundant. In fission yeast, *fbh1Δ* and *srs2Δ* mutations cause a severe growth defect when combined [26], consistent with the idea that both helicases play overlapping roles in HR required for mitotic growth.

Widely conserved FANCM orthologs also suppress crossover during mitosis and/or meiosis in budding yeast, fission yeast, and plants [49]–[51]. ScMph1 and SpFml1 dissociate D-loop structures *in vitro* [50], [52]. Future work should address how cells efficiently utilize these DNA helicases to regulate HR.

### Protection of Rad51 filaments from the anti-recombinase activity of Fbh1

Our *in vitro* analyses indicated that, in the absence of the Swi5-Sfr1 complex, Fbh1 inhibits the DNA strand-exchange reaction via disruption of the Rad51 filament, but not when the Rad51 filament has been activated by the Swi5-Sfr1 complex (Figures 2 and 3). Protection of Rad51 by Swi5-Sfr1 from Fbh1-mediated displacement is very efficient (Figure 2). However, Fbh1-mediated inhibition of DNA strand exchange was not completely restored by Swi5-Sfr1 (Figure 3). Our time-course experiment revealed that protection of Rad51 by Swi5-Sfr1 requires a long incubation time (Figure 3), but the mechanistic details underlying this observation remain unclear.

Heyer and colleagues showed that Rad55-Rad57 stabilizes Rad51-ssDNA filaments against disruption by Srs2 in budding yeast [53]. Therefore, the stabilization of auxiliary factors might be a conserved mechanism for quality control of Rad51 nucleoprotein filaments. In the case of Swi5-Sfr1, other unknown factor(s) might be required *in vivo* for efficient Rad51-mediated strand exchange and for protecting against inhibition by Fbh1.

### Fbh1 stimulates branch migration in the strand-exchange reaction *in vitro*

In this study, we showed that Fbh1 could stimulate the strand-exchange reaction only after the reaction has already started (Figure 3). Although the molecular mechanism remains unclear, it is suggested that Fbh1-Skp1 promotes the strand-exchange reaction of already formed JMs, dependently of its helicase/translocase activity. Fbh1 does not promote the strand-exchange reaction mediated by RecA (Figure S6), indicating that the 3′ end of the displaced strand accessible to Fbh1 is not sufficient for the NC promotion. However, it is still possible that the 3′ end of the displaced strand aids in unwinding of linear dsDNA by Fbh1 only when Rad51 is present. In budding yeast, Rad51 stimulates Srs2 helicase activity [19]; similarly, in fission yeast, Rad51 might also stimulate the Fbh1 helicase. Although there is no direct evidence to support Fbh1 pro-recombinase activity *in vivo*, it is possible that variations in Rad51 filaments specific to the HRR sub-pathway exist, and Fbh1 might promote only recombination on a filament that is competent for the SDSA pathway. Further studies will be needed to address this hypothesis.

Because we could not purify the Rad55-Rad57 complex, we were unable to investigate the effect of Fbh1 on the Rad55-Rad57 recombination reaction *in vitro*. However, the single-DSBR assay revealed that Fbh1 suppresses Rad55-Rad57–mediated CO formation *in vivo*. Based on these observations, we speculate that Fbh1 reduces CO formation by stimulating the Swi5-Sfr1 pathway while simultaneously suppressing the Rad55-Rad57 pathway. In this context, it will be important to determine whether Rad51 filaments are resistant to Fbh1 in the presence of Rad55-Rad57.

### Role of Fbh1 ubiquitin-ligase activity

Rad51 is ubiquitinated by SCF^Fbh1^ *in vitro* (Figure 4). ssDNA prevented ubiquitination of Rad51, implying that Rad51 is not ubiquitinated during HRR repair, when Rad51 forms filaments on ssDNA. Although no currently available evidence directly supports this idea, Rad51 may be ubiquitinated after Rad51 is unloaded from ssDNA either after DNA damage is repaired or when unnecessary Rad51 filaments are formed.

Furthermore, the stability of Rad51 protein in the stationary phase is regulated by Fbh1 (Figure 4D). Previously, we showed that the *fbh1Δ* mutant loses viability in stationary phase, and that deletion of *rad51* or *rad57* suppresses this loss of viability [26]. High levels of Rad51 cause abnormal nuclear division [54], possibly by causing inappropriate recombination. Therefore, we propose that Fbh1 controls the level of Rad51 protein to suppress inappropriate recombination events, and that this interaction might be significant even in the late steps of HR.

Rad51 was mono-ubiquitinated rather than poly-ubiquitinated, raising several related possibilities: (i) ubiquitination of Rad51 is not a signal for protein degradation; (ii) other E3 and/or E4 enzymes are required for polyubiquitination of Rad51 [55]; or (iii) appropriate ubiquitination requires other unknown factor(s). Because Rad51 is phosphorylated in *S. cerevisiae* and human [56]–[60], we favor the third possibility, i.e., that phosphorylation of Rad51 is required for its efficient ubiquitination. In this regard, since the F-box of Fbh1 is essential for mitotic HRR [28], Rad51 ubiquitination by Fbh1 must play an active rather than a passive role, such as one limited to the degradation of Rad51. We attempted to identify the ubiquitination site by mass spectrometry, but the attempt was unsuccessful. The identification of the true ubiquitination site and analysis of the ubiquitination site mutant(s) would provide insight into the biological role(s) of Rad51 ubiquitination.

Two recent studies showed that HsFbh1 is ubiquitinated *in vivo* [43], [45]. This ubiquitination, which depends on PCNA and CRL4^Cdt2^, leads to degradation of HsFbh1 [43]. Therefore, regulation of Fbh1 itself might also be important for genome integrity. Furthermore, HsFbh1 is deregulated in melanocytes, and depletion of HsFbh1 promotes UV-mediated transformation of these cells [61]. Further analysis of Fbh1 function in human cancer cells should increase our understanding of the role this protein plays during tumorigenesis.

**Figure 5:**
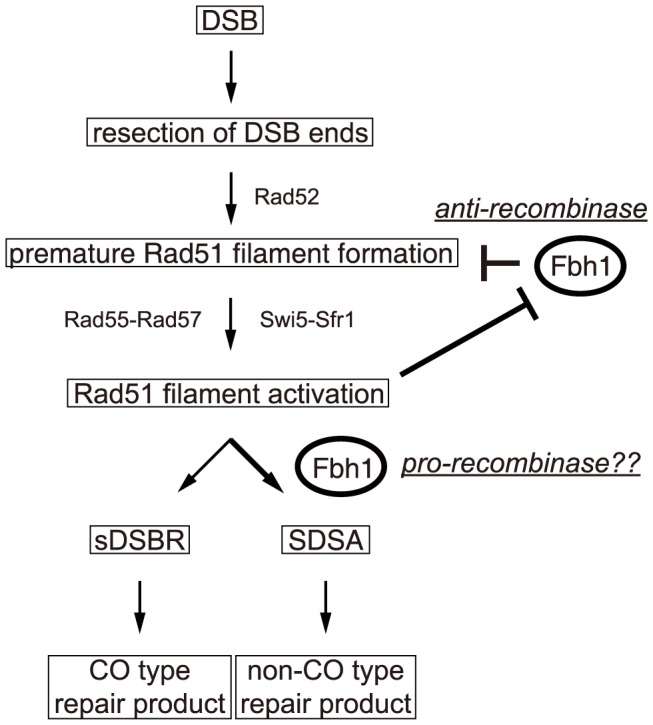
A model for regulation of HRR by Fbh1. After DSB formation, the ends of a DSB are nucleolytically processed to produce recombinogenic DNA with 3′ ss overhangs. Rad51 binds the ssDNA region, an interaction that is mediated by Rad52. Because the resulting Rad51 filament is not yet activated (‘premature’), it is prone to be disrupted by the ‘anti-recombinase’ activity of Fbh1. Swi5-Sfr1 protects the Rad51 filament by activating it. It remains unclear whether Rad55-Rad57 also protects the Rad51 filament. Previously, we proposed that both Rad55-Rad57 and Swi5-Sfr1 sub-pathways are involved in DSBR and synthesis-dependent strand annealing (SDSA), and that Rad55-Rad57 is required for second-end capture (SEC) to form the double-Holliday junction in DSBR [8]. In this study, the results of the single-DSBR assay suggest that Fbh1 suppresses crossover formation, most probably by inhibiting Rad55-Rad57-mediated SEC during DSBR. On the other hand, the *in vitro* data showing Fbh1 stimulation of branch migration in the strand-exchange reaction suggests that Fbh1 might have a pro-recombination role *in vivo*, probably by stimulating the SDSA pathway to generate a non-crossover repair product. This possibility needs to be addressed in future studies. Subsequently, Rad51 would be degraded by SCF^Fbh1^-dependent ubiquitination to prevent re-activation, either after the reaction or during stationary phase.

Taken together, the results of this study demonstrate that Fbh1 plays multiple roles in regulation of recombination (Figure 5). By negatively regulating Rad51 association with ssDNA, Fbh1 would suppress recombination events. Once Swi5-Sfr1 has activated a Rad51 filament, Fbh1 is prevented from exerting its anti-recombinase activity. Once a Rad51 filament is thereby “committed”, Fbh1 is likely to accelerate the strand-exchange reaction *in vivo*. Future studies should address how the Swi5-Sfr1 interaction with Rad51 filaments prevents disruption by Fbh1, as well as the mechanism underlying the strand exchange stimulation by Fbh1. In addition, our analyses also suggest that Fbh1 contributes to regulation of homologous recombination by down-regulating Rad51 levels during stationary phase. Future work should seek to determine why cells require this multi-functional DNA helicase for regulation of homologous recombination.

## Materials and Methods

### Strains, media and growth conditions

The *S. pombe* strains used in this study are shown in Table S4. Standard procedures were used for culture and genetic manipulations [62].

### Site-specific DSBR assay

The assay system and procedures were previously described [38]. Experiments were carried out three to five times for each strain. Detailed PFGE procedures are also described in [38]. The average frequencies of NHEJ/SCC, HRR, and minichromosome loss were calculated as (%Ade^+^Kan^r^), {(%Ade^+^Kan^s^)×[(%GC)+(%CO)+(%BIR type1)]+(%Ade^+^Kan^s^)×[(%LTGC)+(%BIR type2)+(%GC w/CO)]}, and [(%Ade^+^Kan^s^)×(%Minichromosome loss)], respectively.

To determine the copy numbers relative to the parental wild-type strain by quantitative PCR (qPCR), the following primer sets were used: act1-f (5′-GATAATGGCTCTGGTATGTG-3′) and act1-r (5′-CTTTTGTCCCATACCTACCA-3′) for *act1*; ade6-f (5′-CGCATCGACTTCCACAACTCATGCG-3′) and ade6-r (5′-CCGGGAATGGACAGAGAACG-3′) for *ade6*; HOcs-f (5′-CCTAGTTCACAAACAGCCAAAGATTCTC-3′) and HOcs-r (5′-TGTACGGGCGACAGTCACATCATGC-3′) for *HOcs*.

For sequence analysis of the *HOcs* region, genomic DNA was isolated from each Ade^+^Kan^r^ clone and the *HOcs* region was amplified by PCR using rad21-F3435 (5′-AGCACCAACGTACTGATACCTCAAC-3′) and KanMX-R348 (5′-AAGAACCTCAGTGGCAAATCCTAAC-3′). The resultant PCR product was purified from an agarose gel and sequenced using the rad21-F3435 primer.

### Recombinant baculovirus

A recombinant baculovirus expressing *S. pombe* 6His-Fbh1 was constructed using BaculoDirect (Invitrogen) and a pENTR4 derivative, pENTR41-6His-Fbh1. Recombinant baculoviruses expressing *S. pombe* 6His-Skp1, 6His-Pcu1, and 6His-Rbx1 were prepared using the Bac-to-Bac expression system (Invitrogen).

### Expression and purification of Skp1

Insect cells were infected with the 6His-Skp1 baculovirus, and 2×10^9^ cells were harvested from 1 L culture after 72 h. The cells were resuspended in R buffer (20 mM Tris-HCl [pH 8.0], 1 mM EDTA, 1 mM dithiothreitol [DTT], 10% glycerol) containing 300 mM NaCl and homogenized, and the sample was centrifuged at 30,000× g for 30 min. Clarified lysate was directly loaded onto TALON Metal Affinity Resin (Clontech). Bead-bound proteins were eluted in R buffer containing 300 mM NaCl and 300 mM imidazole and lacking DTT and EDTA. The sample was diluted 3-fold with R buffer and loaded onto a HiTrap Heparin column (GE Healthcare). The pass-through fraction was loaded onto a HiTrap Q column (GE Healthcare), and 6His-Skp1 was eluted at ∼550 mM NaCl with a linear gradient of 0.1–1.0 M NaCl in R buffer. Peak fractions were collected and dialyzed against T buffer (20 mM Tris-HCl [pH 7.5], 50% glycerol, 1 mM DTT) containing 300 mM NaCl. The Protein Assay Kit (Bio-Rad), with bovine serum albumin as the standard protein, was used for determination of the protein concentration of Skp1.

### Expression and purification of the Fbh1-Skp1 complex

To express the Fbh1-Skp1 complex, insect cells were co-infected with 6His-Fbh1 and 6His-Skp1 baculoviruses. The ratio of Fbh1 virus to Skp1 virus was determined by measuring the expression levels of these proteins in the host cells. After 72 h, 3.6×10^9^ cells were harvested. The cells were resuspended in R buffer containing 750 mM NaCl and homogenized, and the sample was centrifuged at 30,000× g for 1 h. Clarified lysate was directly loaded onto TALON Metal Affinity Resin. Bead-bound proteins were eluted in R buffer containing 750 mM NaCl and 300 mM imidazole and lacking DTT and EDTA. The sample was diluted 5-fold with R buffer and loaded onto a HiTrap Q column. 6His-Fbh1 and 6His-Skp1 were co-eluted at ∼500 mM NaCl with a linear gradient of 0.1–1.0 M NaCl in R buffer. Eluted fractions were diluted 5-fold with R buffer and loaded onto a HiTrap Heparin column. 6His-Fbh1 and 6His-Skp1 were co-eluted at ∼600 mM NaCl with a linear gradient of 0.1–1.0 M NaCl in R buffer. 6His-Fbh1 and 6His-Skp1 fractions were applied to a HiLoad 16/60 Superdex 200 pg column (GE Healthcare) and developed in R buffer containing 1 M NaCl. Peak fractions were collected, diluted with R buffer, and loaded onto a ResourceQ column (GE Healthcare). 6His-Fbh1 and 6His-Skp1 were co-eluted at ∼600 mM NaCl with a linear gradient of 0.1–1.0 M NaCl in R buffer. Peak fractions were collected and dialyzed against T buffer containing 300 mM NaCl. Concentration of the Fbh1-Skp1 complex was determined as for Skp1 (above), under the assumption that the proteins formed a 1∶1 stoichiometric complex. The complex of 3FLAG6His-Fbh1 and 6His-Skp1 was prepared similarly.

### Purification of the Pcu1-Rbx1 complex

To express the Pcu1-Rbx1 complex, insect cells were co-infected with the 6His-Pcu1 and 6His-Rbx1 baculoviruses. After 72 h, 3.6×10^9^ cells were harvested. The cells were homogenized, and the sample was centrifuged at 30,000× g for 1 h. Clarified lysate was directly loaded onto TALON Metal Affinity Resin. Bead-bound proteins were eluted in R buffer containing 300 mM NaCl and 300 mM imidazole and lacking DTT and EDTA. The sample was diluted 3-fold with R buffer and loaded onto a HiTrap Q column. 6His-Pcu1 and 6His-Rbx1 were co-eluted at ∼500 mM NaCl with a linear gradient of 0.1–1.0 M NaCl in R buffer. Eluted fractions were diluted 5-fold with R buffer and loaded onto a HiTrap Heparin column. 6His-Pcu1 and 6His-Rbx1 were co-eluted at ∼600 mM NaCl with a linear gradient of 0.05–1.0 M NaCl in R buffer. Peak fractions were collected and dialyzed against T buffer containing 300 mM NaCl. Concentration of the Pcu1-Rbx1 complex was determined as for Skp1 (above), under the assumption that the proteins formed a 1∶1 stoichiometric complex.

### Purification of 3HA-ubiquitin, E1, and E2 enzymes

Recombinant *S. pombe* ubiquitin protein was expressed in the *E. coli* BL21-CodonPlus(DE3)-RIPL strain carrying a 3HA-tagged expression plasmid derived from pET11b (Novagen). Expression of 3HA-ubiquitin was induced with 0.5 mM isopropyl-β-D-thiogalactopyranoside (IPTG) at 30°C for 5 h. The cells were collected by centrifugation, resuspended in R buffer containing 300 mM NaCl, and disrupted by sonication; the lysate was clarified by ultracentrifugation. Proteins in the lysate were precipitated by ammonium sulfate fractionation at 40% saturation and centrifuged at 35,000× g for 30 min. The pellet was resuspended in R buffer containing ammonium sulfate at 20% saturation and directly loaded onto a HiTrap Phenyl FF (high sub) column (GE Healthcare). 3HA-ubiquitin was eluted at nearly 0% ammonium sulfate with a linear gradient of 20–0% ammonium sulfate in R buffer. Eluted fractions were dialyzed against R buffer containing 20 mM NaCl. The dialyzed sample was applied to a HiTrap Q column. Eluted 3HA-Ubiquitin was eluted at ∼300 mM NaCl with a linear gradient of 20–600 mM NaCl in R buffer. 3HA-ubiquitin fractions were applied to a HiLoad 16/60 Superdex 200 pg column and developed in R buffer containing 500 mM NaCl. Peak fractions were collected and concentrated using an Amicon Ultra-4 centrifugal device (Millipore) and dialyzed against R buffer containing 100 mM NaCl.

Expression of *S. pombe* 6His-Uba1 was induced with 0.5 mM IPTG at 18°C for 5 h. Harvested cells were disrupted as described above, and clarified lysate was directly loaded on Ni-NTA agarose. Bead-bound proteins were eluted in R buffer containing 300 mM NaCl and 200 mM imidazole and lacking DTT and EDTA. The sample was diluted 3-fold with R buffer and loaded onto a HiTrap Heparin column. The pass-through fraction was loaded directly onto a HiTrap SP column. The subsequent pass-through fraction was loaded onto a HiTrap Q column, and 6His-Uba1 was eluted at ∼300 mM NaCl with a linear gradient of 50–500 mM NaCl in R buffer. Peak fractions were applied to a HiLoad 16/60 Superdex 200 pg column and developed in R buffer containing 1 M NaCl. Peak fractions were collected and dialyzed against R buffer containing 200 mM NaCl.

Expression of *S. pombe* 6His-Ubc4 and 6His-Ubc15 was induced with 0.5 mM IPTG at 30°C for 3 h, respectively. Purification proceeded as described for 6His-Uba1, through the step in which the sample was loaded onto a HiTrap Heparin column. At that stage, 6His-Ubc4 was eluted from the HiTrap Heparin column at ∼300 mM NaCl with a linear gradient of 50–800 mM NaCl in R buffer. Peak fractions were diluted and loaded onto a HiTrap SP column, eluted at ∼200 mM NaCl with a linear gradient of 50–600 mM NaCl in R buffer. 6His-Ubc15 was eluted from the HiTrap Heparin column at ∼200 mM NaCl with a linear gradient of 50–600 mM NaCl in R buffer. Peak fractions were diluted and loaded onto a HiTrap SP column in R buffer containing 50 mM NaCl. The pass-through fraction was loaded directly onto a HiTrap Q column, and 6His-Ubc15 was eluted at ∼250 mM NaCl with a linear gradient of 50–600 mM NaCl in R buffer.

Protein concentrations were determined by measuring absorbance at 280 nm. The following extinction coefficients (ε280) were used: 1.49×10^4^ M^−1^ cm^−1^ for 3HA-Ubiquitin (Ub), 7.61×10^4^ M^−1^ cm^−1^ for 6His-Uba1 (E1), 2.58×10^4^ M^−1^ cm^−1^ for 6His-Ubc4 (E2), and 2.26×10^4^ M^−1^ cm^−1^ for 6His-Ubc15 (E2).

### Purification of Rad51, Swi5-Sfr1, and RPA

Expression and purification of Fbh1-Skp1, Pcu1-Rbx1, Rad51, ubiquitin, Uba1, Ubc4, and Ubc15 are described in Supplemental Experimental Procedures. Rad51, Swi5-Sfr1, and RPA were bacterially expressed and purified as described previously [12], [44].

### Human ubiquitin, E1, and E2 enzymes

Human biotin-ubiquitin, E1, E2 enzymes were purchased from Enzo Life Sciences Inc.

### DNA substrates

DNA substrates used in three-strand exchange reactions were prepared as described previously [12]. A cccDNA of pBluescript SKII, which was used for D-loop analysis, was purified essentially according to the detergent lysis protocol described previously [63]. Synthetic oligonucleotides were purchased from Operon, Ltd. The sequences of the ssDNA oligonucleotides were as follows: F3-d47 (47-mer for ssDNA, dsDNA binding and helicase assay), 5′-AGCTATGACCATGATTACGAATTGCTTGGAATCCTGACGAACTGTAG-3′; F3-d47R (47-mer for dsDNA binding and helicase assay), 5′-CTACAGTTCGTCAGGATTCCAAGCAATTCGTAATCATGGTCATAGCT-3′; d20 (20-mer for helicase assay), 5′-CTACAGTTCGTCAGGATTCC-3′; d22, 5′- AATTCGTGCAGGCATGGTAGCT-3′; 49N2 (49-mer for helicase assay), 5′-AGCTACCATGCCTGCACGAATTCGTATCAGCGTAATCATGGTCATAGCT-3′.

### ATPase assay

These procedures were essentially conducted as described previously [44]. Reaction mixtures (13.5 µl) contained 25 mM Tris-acetate (pH 7.5), 1 mM DTT, 5% glycerol, 3 mM Mg-acetate, 100 mM KCl, and 30 nM Fbh1-Skp1, Fbh1^K301A^-Skp1, or Skp1 alone. In some assays, 10 µM øX174 ssDNA or 10 µM *Apa*LI-linearized øX174 dsDNA were added, as indicated. The reactions were started by adding 1.5 µl of a mixture of [γ-^32^P]ATP and cold ATP (final concentration, 2 mM) at 30°C. Aliquots (2 µl) were taken at various time points and immediately mixed with 4 µl of stop solution (0.5 M EDTA). Samples (1 µl) were subjected to thin-layer chromatography, as described previously [12]. The amounts of ^32^P_i_ and [γ-^32^P]ATP in each spot were determined using a phosphoimager (Fuji BAS2500).

### Gel-shift assay for DNA binding

DNA substrates for dsDNA (F3-d47R+^32^P-labeled F3-d47) and ssDNA (^32^P-labeled F3-d47) binding were as described above. Reaction mixtures contained 10 nM substrate DNA (ssDNA or dsDNA), the indicated concentration of Fbh1-Skp1 proteins, 25 mM Tris-acetate (pH 8.0), 1 mM DTT, 3 mM Mg-acetate, 5% glycerol, and 100 mM KCl. Reactions were incubated at 30°C for 10 min. Products were resolved by electrophoresis on 6% PAGE in TBE buffer. Gels were dried onto DE81 paper, and radiolabeled bands were detected using a Fuji BAS2500 image analyzer.

### Helicase assay

Preparation of 5′-^32^P-labeled substrates with a blunt end, 5′-overhang, or 3′-overhang were previously described [44], [64]. All DNA concentrations are expressed in moles of substrates.

Reaction mixtures (50 µl) containing Fbh1-Skp1 (30 nM), substrate DNA (2 nM), 2 mM ATP, 25 mM Tris-acetate (pH 8.0), 1 mM DTT, 3 mM Mg-acetate, 5% glycerol, and an ATP regeneration system (8 mM creatine phosphate and 8 U ml^−1^ creatine kinase) were incubated at 30°C. At the indicated times, 10 µl of each reaction was collected, stopped with 6× stop buffer containing SDS and proteinase, and incubated for 10 min. Products were resolved by electrophoresis on 12% PAGE in TBE buffer. Gels were dried onto DE81 paper, and radiolabeled bands were detected using a Fuji BAS2500 image analyzer.

### Assays for protein-protein interaction

*In vitro* co-immunoprecipitation assays were performed as follows. Each protein (0.5 µg) as indicated was mixed in 10 µl buffer (25 mM Tris-OAc [pH 7.5], 100 mM KCl, 0.5% NP40, 3 mM Mg-acetate) for 10 min at room temperature followed 20 min at 4°C. To these samples were added M2 FLAG tag antibodies in 90 µl buffer, and the mixtures were incubated for 1 h at 4°C. Dynabeads Protein G (Invitrogen) were then added, and the samples were incubated for 1 h incubation at 4°C. Immunocomplexes were washed three times with 200 µl buffer, and bead-bound proteins were eluted with SDS loading buffer and subjected to 10% SDS-PAGE. Protein complexes were detected by western blotting using anti-Rad51, anti-Sfr1, or anti-M2 antibody (Sigma).

### ssDNA beads assay

ssDNA beads bound to a 608 ntd ssDNA sequence from phiX174 were prepared as described in [65]. The amount of ssDNA attached to the beads was quantified by qPCR. For pull-down experiments, the beads were incubated with Rad51 for 5 min at 37°C, followed by incubation with the Swi5-Sfr1 complex for 5 min at 37°C if required. Beads were washed once, and the resultant Rad51-ssDNA beads were challenged with the Fbh1-Skp1 complex at the indicated times. The buffer used in this assay was 30 mM Tris-HCl (pH 7.5), 1 mM DTT, 100 mM NaCl, 3.5 mM MgCl_2_, 5% glycerol, and 0.25% Tween 20, and an ATP regeneration system (8 mM creatine phosphate and 8 U ml^−1^ creatine kinase). The supernatant and bead fractions were analyzed by SDS-PAGE, and separated proteins were visualized with Coomassie G-250. The quantification of the amount of Rad51 was performed on an LAS-4000 mini (GE Healthcare).

### Three-strand DNA exchange reaction

Three-strand DNA exchange reactions were performed as described in [12]. Fbh1-Skp1 complex was added at the indicated times. Reactions were incubated for 120 min and stopped by addition of a mixture containing 0.9 µl 10% (w/v) SDS, 0.3 µl 0.5 M EDTA, and 0.6 µl 20 mg/ml proteinase K, with a final incubation for 30 min at 37°C. Aliquots were mixed with 6× loading buffer, loaded onto a 1% (w/v) agarose gel, and electrophoresed at 50 V for 2.5 h at room temperature. To visualize DNA bands, gels were stained with SYBR-Gold (Molecular Probes), gel images were captured on an LAS-4000mini, and the intensities of DNA bands were quantified using Multi Gauge (GE healthcare). The percentage of final product was defined as [NC]/([JM]/1.5+[NC]+[ldsDNA])×100, and the percentage of intermediates was defined as ([JM]/1.5)/([JM]/1.5+[NC]+[ldsDNA])×100. In these expressions, the values in square brackets are the intensities of each type of DNA molecule: NC denotes nicked circular dsDNA, and JM denotes joint molecules.

### Ubiquitination assay

For reconstitution of SCF^Fbh1^, 100 nM of Fbh1-Skp1 and Pcu1-Rbx1 were mixed in buffer U (25 mM TrisOAc [pH 7.5], 5 mM Mg/ATP, 1 mM DTT) for 10 min at 37°C. *In vitro* ubiquitination was carried out in buffer U containing 0.1 µM Uba1, 2.5 µM E2, 0.1 µM SCF^Fbh1^, 2.5 µM 3HA-Ub WT, and 1 µM Rad51. After incubation for 30 min at 37°C, the reaction was stopped by addition of 6× SDS-PAGE loading buffer and boiling for 5 min at 95°C. Samples were analyzed by western blotting.

### Analysis of Rad51 and Rad52 protein levels *in vivo*

For investigations of Rad51 and Rad52 protein levels, cells were grown in YES media. To obtain log-phase samples, cells were collected at 0.5×10^7^ cells/ml and treated with 20% of trichloroacetic acid (TCA). For the stationary-phase samples, cells were grown to saturation. After incubation for a further 48 hours, cells were collected and treated with 20% of TCA. Cell lysates were prepared by disruption with glass beads as described in [66], and then analyzed by western blotting.

## Supporting Information

Figure S1 Schematic of genetic assay for HO-induced DSB repair. Parental strain retains a minichromosome Ch16-MG, a derivative of essential Chromosome III. Centromeric regions, the complementary *ade6-M216* and *ade6-M210* heteroalleles were shown by circles and small vertical bars, respectively. Induction of HO endonuclease expression results in a DSB at *HOcs* indicated by a black box on Ch16-MG. Three phenotypes (Ade^+^ G418-resistant (G418^r^), Ade^−^ G418-sensitive (G418^s^), and Ade^+^ G418^s^) can arise following DSB repair. Most segregants with the Ade^+^ G418^r^ phenotype are predicted to arise by NHEJ or SCC. The DSB repair products of G418^s^ segregants were determined by analyzing the chromosomes with pulsed-field gel electrophoresis (PFGE).
(EPS)

Figure S2 Assessment of the NHEJ/SCC population upon HO induction. (A) Primers for determining the copy numbers of the *HOcs*, *ade6*, and *act1* genes. (B) Relative *ade6/act1* ratio, with or without HO induction. Copy numbers of the *ade6* and *act1* genes relative to the parental wild-type strain were determined by quantitative PCR. Then, the *ade6/act1* ratio is calculated as [2× (relative copy number of *ade6*)/(relative copy number of *act1*)]. Each mean and SE was obtained from three independent experiments. (C) Relative *HOcs/act1* ratio with or without HO induction. Copy numbers of the *HOcs* and *act1* genes relative to the parental wild-type strain were determined. The *HOcs/act1* ratio was calculated as [(relative copy number of *ade6*)/(relative copy number of *act1*)]. Each mean and SE was obtained from three independent experiments. (D) Summary of sequence analysis of the *HOcs* region from 48 independent clones. No NHEJ clone was observed. Two clones in the wild type strain and the *fbh1Δ* mutant showed heterogeneous sequence, which are thought to be independent on NHEJ.
(EPS)

Figure S3 Initial characterization of Fbh1-Skp1 complex. (A) SDS-PAGE analysis of recombinant proteins. (B) ATPase assay of Fbh1-Skp1 in the presence of DNA. (C) ATPase assays with Fbh1 K301A-Skp1 and Skp1. (D) DNA-binding assay. (E) DNA helicase assay.
(EPS)

Figure S4 Stability of Rad51 filament challenged by Fbh1 after long incubation. (A) Schematic diagram of the ssDNA-bead assay with long incubation. Note that this experiment contains no washing step before addition of Fbh1. After preparation of ssDNA beads, Rad51 was incubated with the beads to form Rad51-ssDNA filaments. Subsequently, Fbh1 was added before or after Swi5-Sfr1 addition. Buffer conditions and protein concentration were the same as those in the three-strand exchange reaction, except that 0.05% NP-40 was added here. (B) Gel image of the assay. After incubation for 60 min, beads were washed twice and the bead-bound fraction was analyzed by SDS-PAGE and visualized by CBB-G250 staining. The gel was 9% (29∶1 acrylamide∶bisacrylamide). (C) Intensity of Rad51 in panel B was quantitated, and percentage reduction at each reaction was calculated as [(Rad51 amount on Beads in the presence of Fbh1)/(Rad51 amount on Beads in the absence of Fbh1)×100]. Each mean and SE was obtained from three independent experiments.
(EPS)

Figure S5 Fbh1 does not stimulate Rad51-mediated strand-exchange reaction in the absence of Swi5-Sfr1. (A) Schematic of the three-strand exchange reactions. Fbh1 (90 nM) was added at the indicated step, (i)–(viii). (B) Gel image of three-strand exchange reactions.
(EPS)

Figure S6 Fbh1 inhibits RecA-mediated strand-exchange reaction, but does not stimulate. (A) Schematic of the three-strand exchange reactions by *E. coli* RecA. Fbh1 (90 nM) was added at the indicated step, (a)–(c). (B) Gel image of three-strand exchange reactions. (C) Quantitation of results in panel B. Each mean value and SE was obtained from three independent experiments.
(EPS)

Figure S7 *In vitro* ubiquitination assay. (A) *In vitro* immunoprecipitation. FLAG-His–tagged Fbh1-Skp1 was immunoprecipitated with anti-FLAG antibody. Precipitated proteins were analyzed by western blotting using anti-FLAG (upper) and anti-Rad51 or anti-Sfr1 (lower) antibodies. (B) SpRad51 is ubiquitinated *in vitro* by human ubiquitin, E1, E2, and SpSCF^Fbh1^ E3 ligase complex. The reaction mixture was analyzed by western blotting with affinity-purified anti-Rad51 antibody. (C) The same membrane used in (B) was stripped and re-probed with streptavidin-HRP to detect ubiquitin molecules.
(EPS)

Figure S8 Unrooted phylogenetic tree of Ubc proteins in budding yeast (Sc), fission yeast (Sp), and human (Hs). A multiple alignment was constructed using the Clustal X program [67]. On the basis of the alignment, an unrooted phylogenic tree was constructed by the neighbor-joining method [68] using the genetic distances. The reference sequences of the Ubc proteins used in this study were obtained from the following databases: Saccharomyces Genome Database (http://www.yeastgenome.org), Pombase (http://www.pombase.org), and the Human Protein Reference Database (http://www.hprd.org/index_html).
(EPS)

Table S1 Genetic determination of site-specific DSB-induced marker loss.
(DOC)

Table S2 PFGE analysis of Ade^+^ G418^s^ segregants.
(DOC)

Table S3 PFGE analysis of Ade^−^ G418^s^ segregants.
(DOC)

Table S4 *S. pombe* strains used in this study.
(DOC)

## References

[1] New JH, Sugiyama T, Zaitseva E, Kowalczykowski SC (1998) Rad52 protein stimulates DNA strand exchange by Rad51 and replication protein A. Nature 391: 407–410. 9450760 10.1038/34950

[2] Shinohara A, Ogawa T (1998) Stimulation by Rad52 of yeast Rad51-mediated recombination. Nature 391: 404–407. 9450759 10.1038/34943

[3] Sung P (1997) Function of yeast Rad52 protein as a mediator between replication protein A and the Rad51 recombinase. J Biol Chem 272: 28194–28197. 9353267 10.1074/jbc.272.45.28194

[4] West SC, Benson FE, Baumann P (1998) Synergistic actions of Rad51 and Rad52 in recombination and DNA repair. Nature 391: 401–404. 9450758 10.1038/34937

[5] Sung P (1997) Yeast Rad55 and Rad57 proteins form a heterodimer that functions with replication protein A to promote DNA strand exchange by Rad51 recombinase. Genes Dev 11: 1111–1121. 9159392 10.1101/gad.11.9.1111

[6] San Filippo J, Chi P, Sehorn MG, Etchin J, Krejci L, et al (2006) Recombination mediator and Rad51 targeting activities of a human BRCA2 polypeptide. J Biol Chem 281: 11649–11657. 16513631 10.1074/jbc.M601249200 PMC2077811

[7] Akamatsu Y, Dziadkowiec D, Ikeguchi M, Shinagawa H, Iwasaki H (2003) Two different Swi5-containing protein complexes are involved in mating-type switching and recombination repair in fission yeast. Proc Natl Acad Sci USA 100: 15770–15775. 14663140 10.1073/pnas.2632890100 PMC307643

[8] Akamatsu Y, Tsutsui Y, Morishita T, Siddique MSP, Kurokawa Y, et al (2007) Fission yeast Swi5/Sfr1 and Rhp55/Rhp57 differentially regulate Rhp51-dependent recombination outcomes. EMBO J 26: 1352–1362. 17304215 10.1038/sj.emboj.7601582 PMC1817630

[9] Akamatsu Y, Jasin M (2010) Role for the mammalian Swi5-Sfr1 complex in DNA strand break repair through homologous recombination. PLoS Genet 6: e1001160. 20976249 10.1371/journal.pgen.1001160 PMC2954829

[10] Hayase A, Takagi M, Miyazaki T, Oshiumi H, Shinohara M, et al (2004) A protein complex containing Mei5 and Sae3 promotes the assembly of the meiosis-specific RecA homolog Dmc1. Cell 119: 927–940. 15620352 10.1016/j.cell.2004.10.031

[11] Tsubouchi H, Roeder GS (2004) The budding yeast Mei5 and Sae3 proteins act together with Dmc1 during meiotic recombination. Genetics 168: 1219–1230. 15579681 10.1534/genetics.103.025700 PMC1448777

[12] Haruta N, Kurokawa Y, Murayama Y, Akamatsu Y, Unzai S, et al (2006) The Swi5-Sfr1 complex stimulates Rhp51/Rad51- and Dmc1-mediated DNA strand exchange in vitro. Nat Struct Mol Biol 13: 823–830. 16921379 10.1038/nsmb1136

[13] Rong L, Palladino F, Aguilera A, Klein H (1991) The hyper-gene conversion *hpr5-1* mutation of *Saccharomyces cerevisiae* is an allele of the *SRS2/RADH* gene. Genetics 127: 75–85. 1849857 10.1093/genetics/127.1.75 PMC1204314

[14] Schiestl RH, Prakash S, Prakash L (1990) The *SRS2* Suppressor of *rad6* Mutations of *Saccharomyces cerevisiae* Acts by Channeling DNA Lesions Into the *RAD52* DNA Repair Pathway. Genetics 124: 817–831. 2182387 10.1093/genetics/124.4.817 PMC1203974

[15] Aboussekhra A, Chanet R, Zgaga Z, Cassier-Chauvat C, Heude M, et al (1989) *RADH*, a gene of *Saccharomyces cerevisiae* encoding a putative DNA helicase involved in DNA repair. Characteristics of *radH* mutants and sequence of the gene. Nucleic Acids Res 17: 7211–7219. 2552405 10.1093/nar/17.18.7211 PMC334801

[16] Marini V, Krejci L (2010) Srs2: the “Odd-Job Man” in DNA repair. DNA Repair 9: 268–275. 20096651 10.1016/j.dnarep.2010.01.007 PMC2845805

[17] Krejci L, van Komen S, Li Y, Villemain J, Reddy MS, et al (2003) DNA helicase Srs2 disrupts the Rad51 presynaptic filament. Nature 423: 305–309. 12748644 10.1038/nature01577

[18] Veaute X, Jeusset J, Soustelle C, Kowalczykowski SC, Le Cam E, et al (2003) The Srs2 helicase prevents recombination by disrupting Rad51 nucleoprotein filaments. Nature 423: 309–312. 12748645 10.1038/nature01585

[19] Dupaigne P, Le Breton C, Fabre F, Gangloff S, Le Cam E, et al (2008) The Srs2 helicase activity is stimulated by Rad51 filaments on dsDNA: implications for crossover incidence during mitotic recombination. Mol Cell 29: 243–254. 18243118 10.1016/j.molcel.2007.11.033

[20] Moldovan G-L, Dejsuphong D, Petalcorin MIR, Hofmann K, Takeda S, et al (2012) Inhibition of homologous recombination by the PCNA-interacting protein PARI. Mol Cell 45: 75–86. 22153967 10.1016/j.molcel.2011.11.010 PMC3267324

[21] Wu L, Hickson ID (2003) The Bloom's syndrome helicase suppresses crossing over during homologous recombination. Nature 426: 870–874. 14685245 10.1038/nature02253

[22] Ira G, Malkova A, Liberi G, Foiani M, Haber JE (2003) Srs2 and Sgs1-Top3 suppress crossovers during double-strand break repair in yeast. Cell 115: 401–411. 14622595 10.1016/s0092-8674(03)00886-9 PMC4493758

[23] Bugreev DV, Yu X, Egelman EH, Mazin AV (2007) Novel pro- and anti-recombination activities of the Bloom's syndrome helicase. Genes Dev 21: 3085–3094. 18003860 10.1101/gad.1609007 PMC2081975

[24] Hu Y, Raynard S, Sehorn MG, Lu X, Bussen W, et al (2007) RECQL5/Recql5 helicase regulates homologous recombination and suppresses tumor formation via disruption of Rad51 presynaptic filaments. Genes Dev 21: 3073–3084. 18003859 10.1101/gad.1609107 PMC2081974

[25] Park JS, Choi E, Lee SH, Lee C, Seo YS (1997) A DNA helicase from *Schizosaccharomyces pombe* stimulated by single-stranded DNA-binding protein at low ATP concentration. J Biol Chem 272: 18910–18919. 9228070 10.1074/jbc.272.30.18910

[26] Morishita T, Furukawa F, Sakaguchi C, Toda T, Carr AM, et al (2005) Role of the *Schizosaccharomyces pombe* F-Box DNA helicase in processing recombination intermediates. Mol Cell Biol 25: 8074–8083. 16135799 10.1128/MCB.25.18.8074-8083.2005 PMC1234317

[27] Osman F, Dixon J, Barr AR, Whitby MC (2005) The F-Box DNA helicase Fbh1 prevents Rhp51-dependent recombination without mediator proteins. Mol Cell Biol 25: 8084–8096. 16135800 10.1128/MCB.25.18.8084-8096.2005 PMC1234329

[28] Sakaguchi C, Morishita T, Shinagawa H, Hishida T (2008) Essential and distinct roles of the F-box and helicase domains of Fbh1 in DNA damage repair. BMC Mol Biol 9: 27. 18312697 10.1186/1471-2199-9-27 PMC2294136

[29] Fugger K, Mistrik M, Danielsen JR, Dinant C, Falck J, et al (2009) Human Fbh1 helicase contributes to genome maintenance via pro- and anti-recombinase activities. J Cell Biol 186: 655–663. 19736316 10.1083/jcb.200812138 PMC2742184

[30] Laulier C, Cheng A, Huang N, Stark JM (2010) Mammalian Fbh1 is important to restore normal mitotic progression following decatenation stress. DNA Repair 9: 708–717. 20457012 10.1016/j.dnarep.2010.03.011 PMC2883650

[31] Okamoto S-Y, Sato M, Toda T, Yamamoto M (2012) SCF Ensures Meiotic Chromosome Segregation Through a Resolution of Meiotic Recombination Intermediates. PLoS ONE 7: e30622. 22292001 10.1371/journal.pone.0030622 PMC3264600

[32] Kohzaki M, Hatanaka A, Sonoda E, Yamazoe M, Kikuchi K, et al (2007) Cooperative roles of vertebrate Fbh1 and Blm DNA helicases in avoidance of crossovers during recombination initiated by replication fork collapse. Mol Cell Biol 27: 2812–2820. 17283053 10.1128/MCB.02043-06 PMC1899948

[33] Lorenz A, Osman F, Folkyte V, Sofueva S, Whitby MC (2009) Fbh1 limits Rad51-dependent recombination at blocked replication forks. Mol Cell Biol 29: 4742–4756. 19546232 10.1128/MCB.00471-09 PMC2725720

[34] Sun W, Lorenz A, Osman F, Whitby MC (2011) A failure of meiotic chromosome segregation in a *fbh1Δ* mutant correlates with persistent Rad51-DNA associations. Nucleic Acids Res 39: 1718–1731. 21149262 10.1093/nar/gkq977 PMC3061084

[35] Kim J, Kim J-H, Lee S-H, Kim D-H, Kang H-Y, et al (2002) The novel human DNA helicase hFBH1 is an F-box protein. J Biol Chem 277: 24530–24537. 11956208 10.1074/jbc.M201612200

[36] Kim J-H, Kim J, Kim D-H, Ryu G-H, Bae S-H, et al (2004) SCF^hFBH1^ can act as helicase and E3 ubiquitin ligase. Nucleic Acids Res 32: 2287–2297. 15118074 10.1093/nar/gkh534 PMC419438

[37] Lawrence CL, Jones N, Wilkinson CRM (2009) Stress-induced phosphorylation of *S. pombe* Atf1 abrogates its interaction with F box protein Fbh1. Curr Biol 19: 1907–1911. 19836238 10.1016/j.cub.2009.09.044

[38] Prudden J, Evans JS, Hussey SP, Deans B, O'Neill P, et al (2003) Pathway utilization in response to a site-specific DNA double-strand break in fission yeast. EMBO J 22: 1419–1430. 12628934 10.1093/emboj/cdg119 PMC151045

[39] Hope JC, Mense SM, Jalakas M, Mitsumoto J, Freyer GA (2006) Rqh1 blocks recombination between sister chromatids during double strand break repair, independent of its helicase activity. Proc Natl Acad Sci USA 103: 5875–5880. 16595622 10.1073/pnas.0601571103 PMC1458666

[40] FBH1 helicase disrupts RAD51 filaments in vitro and modulates homologous recombination in mammalian cells (2013) FBH1 helicase disrupts RAD51 filaments *in vitro* and modulates homologous recombination in mammalian cells. J Biol Chem 288: 34168–34180. 24108124 10.1074/jbc.M113.484493 PMC3837158

[41] Wang SW, Goodwin A, Hickson ID, Norbury CJ (2001) Involvement of *Schizosaccharomyces pombe* Srs2 in cellular responses to DNA damage. Nucleic Acids Res 29: 2963–2972. 11452021 10.1093/nar/29.14.2963 PMC55813

[42] Robert T, Dervins D, Fabre F, Gangloff S (2006) Mrc1 and Srs2 are major actors in the regulation of spontaneous crossover. EMBO J 25: 2837–2846. 16724109 10.1038/sj.emboj.7601158 PMC1500851

[43] Bacquin A, Pouvelle C, Siaud N, Perderiset M, Salomé-Desnoulez S, et al (2013) The helicase FBH1 is tightly regulated by PCNA via CRL4(Cdt2)-mediated proteolysis in human cells. Nucleic Acids Res 41: 6501–6513. 23677613 10.1093/nar/gkt397 PMC3711418

[44] Kurokawa Y, Murayama Y, Haruta-Takahashi N, Urabe I, Iwasaki H (2008) Reconstitution of DNA strand exchange mediated by Rhp51 recombinase and two mediators. PLoS Biol 6: e88. 18416603 10.1371/journal.pbio.0060088 PMC2292753

[45] Masuda-Ozawa T, Hoang T, Seo Y-S, Chen L-F, Spies M (2013) Single-molecule sorting reveals how ubiquitylation affects substrate recognition and activities of FBH1 helicase. Nucleic Acids Res 41: 3576–3587. 23393192 10.1093/nar/gkt056 PMC3616717

[46] Skaar JR, Pagan JK, Pagano M (2013) Mechanisms and function of substrate recruitment by F-box proteins. Nat Rev Mol Cell Biol 14: 369–381. 23657496 10.1038/nrm3582 PMC3827686

[47] Kus BM, Caldon CE, Andorn-Broza R, Edwards AM (2004) Functional interaction of 13 yeast SCF complexes with a set of yeast E2 enzymes in vitro. Proteins 54: 455–467. 14747994 10.1002/prot.10620

[48] Chiolo I, Saponaro M, Baryshnikova A, Kim J-H, Seo Y-S, et al (2007) The human F-Box DNA helicase FBH1 faces *Saccharomyces cerevisiae* Srs2 and postreplication repair pathway roles. Mol Cell Biol 27: 7439–7450. 17724085 10.1128/MCB.00963-07 PMC2169053

[49] Sun W, Nandi S, Osman F, Ahn JS, Jakovleska J, et al (2008) The FANCM ortholog Fml1 promotes recombination at stalled replication forks and limits crossing over during DNA double-strand break repair. Mol Cell 32: 118–128. 18851838 10.1016/j.molcel.2008.08.024 PMC2581491

[50] Lorenz A, Osman F, Sun W, Nandi S, Steinacher R, et al (2012) The fission yeast FANCM ortholog directs non-crossover recombination during meiosis. Science 336: 1585–1588. 22723423 10.1126/science.1220111 PMC3399777

[51] Crismani W, Girard C, Froger N, Pradillo M, Santos JL, et al (2012) FANCM limits meiotic crossovers. Science 336: 1588–1590. 22723424 10.1126/science.1220381

[52] Prakash R, Satory D, Dray E, Papusha A, Scheller J, et al (2009) Yeast Mph1 helicase dissociates Rad51-made D-loops: implications for crossover control in mitotic recombination. Genes Dev 23: 67–79. 19136626 10.1101/gad.1737809 PMC2632165

[53] Liu J, Renault L, Veaute X, Fabre F, Stahlberg H, et al (2011) Rad51 paralogues Rad55-Rad57 balance the antirecombinase Srs2 in Rad51 filament formation. Nature 479: 245–248. 22020281 10.1038/nature10522 PMC3213327

[54] Kim WJ, Lee H, Park EJ, Park JK, Park SD (2001) Gain- and loss-of-function of Rhp51, a Rad51 homolog in fission yeast, reveals dissimilarities in chromosome integrity. Nucleic Acids Res 29: 1724–1732. 11292845 10.1093/nar/29.8.1724 PMC31306

[55] Metzger MB, Weissman AM (2010) Working on a chain: E3s ganging up for ubiquitylation. Nat Cell Biol 12: 1124–1126. 21124306 10.1038/ncb1210-1124

[56] Yuan ZM (1998) Regulation of Rad51 Function by c-Abl in Response to DNA Damage. Journal of Biological Chemistry 273: 3799–3802. 9461559 10.1074/jbc.273.7.3799

[57] Chen G, Yuan SS, Liu W, Xu Y, Trujillo K, et al (1999) Radiation-induced assembly of Rad51 and Rad52 recombination complex requires ATM and c-Abl. J Biol Chem 274: 12748–12752. 10212258 10.1074/jbc.274.18.12748

[58] Slupianek A, Schmutte C, Tombline G, Nieborowska-Skorska M, Hoser G, et al (2001) BCR/ABL regulates mammalian RecA homologs, resulting in drug resistance. Mol Cell 8: 795–806. 11684015 10.1016/s1097-2765(01)00357-4

[59] Flott S, Kwon Y, Pigli YZ, Rice PA, Sung P, et al (2011) Regulation of Rad51 function by phosphorylation. EMBO Rep 12: 833–839. 21738226 10.1038/embor.2011.127 PMC3147262

[60] Yata K, Lloyd J, Maslen S, Bleuyard J-Y, Skehel M, et al (2012) Plk1 and CK2 Act in Concert to Regulate Rad51 during DNA Double Strand Break Repair. Mol Cell 45: 371–383. 22325354 10.1016/j.molcel.2011.12.028 PMC3280358

[61] Jeong Y-T, Rossi M, Cermak L, Saraf A, Florens L, et al (2013) FBH1 promotes DNA double-strand breakage and apoptosis in response to DNA replication stress. J Cell Biol 200: 141–149. 23319600 10.1083/jcb.201209002 PMC3549964

[62] Moreno S, Klar A, Nurse P (1991) Molecular genetic analysis of fission yeast *Schizosaccharomyces pombe* . Meth Enzymol 194: 795–823. 2005825 10.1016/0076-6879(91)94059-l

[63] Cunningham RP, DasGupta C, Shibata T, Radding CM (1980) Homologous pairing in genetic recombination: recA protein makes joint molecules of gapped circular DNA and closed circular DNA. Cell 20: 223–235. 7388943 10.1016/0092-8674(80)90250-0

[64] Hishida T, Han Y-W, Shibata T, Kubota Y, Ishino Y, et al (2004) Role of the Escherichia coli RecQ DNA helicase in SOS signaling and genome stabilization at stalled replication forks. Genes Dev 18: 1886–1897. 15289460 10.1101/gad.1223804 PMC517408

[65] Furuya K, Miyabe I, Tsutsui Y, Paderi F, Kakusho N, et al (2010) DDK phosphorylates checkpoint clamp component Rad9 and promotes its release from damaged chromatin. Mol Cell 40: 606–618. 21095590 10.1016/j.molcel.2010.10.026

[66] A phosphatase complex that dephosphorylates gammaH2AX regulates DNA damage checkpoint recovery (2006) A phosphatase complex that dephosphorylates gammaH2AX regulates DNA damage checkpoint recovery. 439: 497–501. 10.1038/nature04384 16299494

[67] Larkin MA, Blackshields G, Brown NP, Chenna R, McGettigan PA, et al (2007) Clustal W and Clustal X version 2.0. Bioinformatics 23: 2947–2948. 17846036 10.1093/bioinformatics/btm404

[68] Saitou N, Nei M (1987) The neighbor-joining method: a new method for reconstructing phylogenetic trees. Mol Biol Evol 4: 406–425. 3447015 10.1093/oxfordjournals.molbev.a040454

