# Supplementary material for: Multiple Regulation of Rad51-Mediated Homologous Recombination by Fission Yeast Fbh1
Source: PLoS Genet. 2014 Aug 28;10(8):e1004542. doi: 10.1371/journal.pgen.1004542 (PMC4148199; doi:10.1371/journal.pgen.1004542)
Supplement: Table S1 — Genetic determination of site-specific DSB-induced marker loss. (DOC) [file pgen.1004542.s009.doc]

**Table S1**

Genetic determination of site-specific DSB-induced marker loss

| Strain | Time  (h) | HO-  induction | Total  scored | % Ade^+^G418^r^ | % Ade^+^G418^s^ | % Ade^-^G418^s^ | DSB-induced Ade^+^G418^s^ | DSB-induced Ade^-^G418^s^ |
| --- | --- | --- | --- | --- | --- | --- | --- | --- |
| ***wild-type*** | 0 | - | 9,714 | 96.7 ± 1.2 | 3.1 ± 1.2 | 0.11 ± 0.0 |  |  |
|  | 48 | - | 12,499 | 96.7 ± 1.1 | 2.9 ± 1.1 | 0.39 ± 0.1 |  |  |
|  | 48 | + | 11,836 | 10.3 ± 2.0 | 65.3 ± 2.4 | 24.4 ± 1.4 | 62.9 ± 1.5 | 23.9 ± 1.4 |
| ***rad51∆* ^a^** | 0 | - | 3,208 | 98.4 ± 0.3 | 0.2 ± 0.1 | 1.4 ± 0.3 |  |  |
|  | 48 | - | 3,197 | 92.1 ± 0.8 | 0.1 ± 0.1 | 7.8 ± 0.8 |  |  |
|  | 48 | + | 2,727 | 16.7 ± 0.7 | 5.0 ± 0.7 | 78.3 ± 1.4 | 4.9 ± 0.6 | 70.5 ± 1.8 |
| ***swi5∆* ^a^** | 0 | - | 4,424 | 99.2 ± 0.4 | 0.6 ± 0.3 | 0.2 ± 0.1 |  |  |
|  | 48 | - | 5,679 | 98.4 ± 0.5 | 0.9 ± 0.4 | 0.7 ± 0.2 |  |  |
|  | 48 | + | 7,150 | 33.1 ± 2.7 | 23.9 ± 1.5 | 43.0 ± 1.8 | 23.0 ± 1.5 | 42.4 ± 1.9 |
| ***sfr1∆* ^a^** | 0 | - | 2,697 | 99.5 ± 0.2 | 0.3 ± 0.2 | 0.2 ± 0.1 |  |  |
|  | 48 | - | 3,017 | 99.4 ± 0.2 | 0.4 ± 0.2 | 0.2 ± 0.1 |  |  |
|  | 48 | + | 4,840 | 25.5 ± 3.9 | 28.0 ± 1.9 | 46.5 ± 3.6 | 27.6 ± 1.8 | 46.3 ± 3.6 |
| ***rad57∆* ^a^** | 0 | - | 4,485 | 96.0 ± 0.6 | 1.1 ± 0.4 | 2.9 ± 0.3 |  |  |
|  | 48 | - | 4,445 | 91.6 ± 1.5 | 1.1± 0.4 | 7.3±1.2 |  |  |
|  | 48 | + | 4,564 | 19.8 ± 3.5 | 9.8 ± 0.8 | 70.4 ± 3.4 | 8.8 ± 0.8 | 63.1 ± 4.4 |
| ***rqh1∆*** | 0 | - | 8,430 | 96.8 ± 0.8 | 2.0 ± 0.5 | 1.1 ± 0.3 |  |  |
|  | 48 | - | 8,020 | 96.1 ± 1.8 | 1.9 ± 0.9 | 2.0 ± 1.0 |  |  |
|  | 48 | + | 4,994 | 70.4 ± 1.7 | 12.0 ± 1.0 | 17.5 ± 0.7 | 10.2 ± 1.0 | 15.5 ± 0.9 |
| ***srs2∆*** | 0 | - | 5,709 | 97.4 ± 1.7 | 2.1 ± 1.4 | 0.5 ± 0.3 |  |  |
|  | 48 | - | 6,213 | 96.4 ± 2.2 | 2.7 ± 1.8 | 0.9 ± 0.4 |  |  |
|  | 48 | + | 4,984 | 42.7 ± 2.3 | 42.5 ± 1.0 | 14.8 ± 1.8 | 39.8 ± 2.5 | 13.9 ± 2.2 |
| ***fbh1∆*** | 0 | - | 5,794 | 98.3 ± 1.4 | 1.3 ± 1.1 | 0.4 ± 0.3 |  |  |
|  | 48 | - | 4,446 | 97.6 ± 1.4 | 1.7 ± 1.2 | 0.7 ± 0.3 |  |  |
|  | 48 | + | 4,818 | 43.4 ± 3.3 | 51.6 ± 3.4 | 4.9 ± 1.9 | 49.9 ± 2.6 | 3.9 ± 3.0 |
| ***rad51∆ fbh1∆*** | 0 | - | 5,652 | 96.3 ± 1.8 | 0.2 ± 0.2 | 3.5 ± 1.6 |  |  |
|  | 48 | - | 4,744 | 92.1 ± 3.2 | 0.4 ± 0.3 | 7.5 ± 2.9 |  |  |
|  | 48 | + | 3,652 | 17.5 ± 2.0 | 5.2 ± 1.3 | 77.3 ± 3.3 | 4.8 ± 1.5 | 69.7 ± 2.3 |
| ***swi5∆ fbh1∆*** | 0 | - | 6,757 | 97.1 ± 2.3 | 2.3 ± 1.8 | 0.6 ± 0.6 |  |  |
|  | 48 | - | 4,973 | 96.6 ± 2.7 | 2.3 ± 2.0 | 1.1 ± 0.8 |  |  |
|  | 48 | + | 6,589 | 32.2 ± 7.6 | 61.0 ± 7.0 | 6.7 ± 1.6 | 58.7 ± 6.8 | 5.6 ± 1.4 |
| ***sfr1∆ fbh1∆*** | 0 | - | 6,810 | 96.4 ± 0.9 | 3.3 ± 0.8 | 0.3 ± 0.1 |  |  |
|  | 48 | - | 7,070 | 95.3 ± 1.3 | 4.1 ± 1.2 | 0.5 ± 0.2 |  |  |
|  | 48 | + | 8,327 | 33.1 ± 2.7 | 60.6 ± 2.9 | 6.2 ± 0.7 | 56.5 ± 3.0 | 5.7 ± 0.6 |
| ***rad57∆ fbh1∆*** | 0 | - | 6,926 | 96.6 ± 0.8 | 2.4 ± 1.1 | 1.1 ± 0.4 |  |  |
|  | 48 | - | 6,099 | 96.3 ± 0.4 | 2.4 ± 0.7 | 1.2 ± 0.4 |  |  |
|  | 48 | + | 7,324 | 56.9 ± 1.6 | 35.3 ± 0.7 | 7.7 ± 0.9 | 32.9 ± 1.1 | 6.5 ± 0.5 |

For each genetic background, independent assays were carried out at least three times. The average value for each time point and the standard errors between the independent experiments are shown. The percentages of DSB-induced Ade^+^G418^S^ and Ade^-^G418^S^ colonies were calculated as [%Ade^+^G418^S^ (48h +induction) – % Ade^+^G418^S^ (48h -induction)] and [%Ade^-^G418^S^ (48h +induction) – %Ade^-^G418^S^ (48h -induction)], respectively.

^a^Data were quoted from Akamatsu et al. (2007) [8].
