# Supplementary material for: Multiple Regulation of Rad51-Mediated Homologous Recombination by Fission Yeast Fbh1
Source: PLoS Genet. 2014 Aug 28;10(8):e1004542. doi: 10.1371/journal.pgen.1004542 (PMC4148199; doi:10.1371/journal.pgen.1004542)
Supplement: Table S2 — PFGE analysis of Ade+ G418s segregants. (DOC) [file pgen.1004542.s010.doc]

**Table S2**

PFGE analysis of Ade^+^ G418^s^ segregants

| Strain | Total analyzed | GC | CO | BIR type1 | Unknown |
| --- | --- | --- | --- | --- | --- |
| ***wild-type*** | 90 | 78 (86.7 %) | 7 (7.8 %) | 1 (1.1 %) | 4 (4.4 %) |
| ***rad51∆* ^a^** | 105 | 105 (100 %) | 0 (0 %) | 0 (0 %) | 0 (0 %) |
| ***swi5∆* ^a^** | 102 | 93 (91.2 %) | 8 (7.8 %) | 1 (1.0 %) | 0 (0 %) |
| ***sfr1∆* ^a^** | 102 | 97 (95.1 %) | 5 (4.9 %) | 0 (0 %) | 0 (0 %) |
| ***rad57∆* ^a^** | 101 | 98 (97.0 %) | 0 (0 %) | 0 (0 %) | 3 (3.0 %) |
| ***rqh1∆*** | 90 | 42 (46.7 %) | 45 (50.0 %) | 3 (3.3 %) | 0 (0 %) |
| ***srs2∆*** | 90 | 71 (78.9 %) | 16 (17.8 %) | 0 (0 %) | 3 (3.3 %) |
| ***fbh1∆*** | 90 | 50 (55.6 %) | 38 (42.2 %) | 0 (0 %) | 2 (2.2 %) |
| ***rad51∆ fbh1∆*** | 90 | 89 (98.9 %) | 0 (0 %) | 1 (1.1 %) | 0 (0 %) |
| ***swi5∆ fbh1∆*** | 120 | 89 (74.2 %) | 28 (23.3 %) | 2 (1.7 %) | 1 (0.8 %) |
| ***sfr1∆ fbh1∆*** | 120 | 78 (65.0 %) | 31 (25.8 %) | 4 (3.3 %) | 7 (5.8 %) |
| ***rad57∆ fbh1∆*** | 90 | 63 (70.0 %) | 19 (21.1 %) | 2 (2.2 %) | 2 (2.2 %) |

For each genetic background, each assay was carried out independently at least three times, and chromosomal DNA from about 30 colonies was subjected to PFGE. Numbers in parentheses indicate the frequencies among all Ade^+^G418^s^ colonies analyzed for each strain.

^a^ Data are from Akamatsu et al. (2007) [8].

GC, gene conversion; CO, crossover; BIR, break induced replication; PGFE, pulsed-field gel electrophoresis
