# Supplementary material for: Multiple Regulation of Rad51-Mediated Homologous Recombination by Fission Yeast Fbh1
Source: PLoS Genet. 2014 Aug 28;10(8):e1004542. doi: 10.1371/journal.pgen.1004542 (PMC4148199; doi:10.1371/journal.pgen.1004542)
Supplement: Table S3 — PFGE analysis of Ade− G418s segregants. (DOC) [file pgen.1004542.s011.doc]

**Table S3**

PFGE analysis of Ade^-^ G418^s^ segregants

| Strain | Total analyzed | Minichromosome loss | Long-tract GC  (LTGC) | BIR type 2 | Two-marker GC w/ CO | Unknown |
| --- | --- | --- | --- | --- | --- | --- |
| ***wild-type*** | 90 | 51 (56.7 %) | 35 (38.9 %) | 2 (2.2 %) | 2 (2.2 %) | 0 (0 %) |
| ***rad51∆* ^a^** | 102 | 83 (81.3 %) | 17 (16.7 %) | 1 (1.0 %) | 1 (1.0 %) | 0 (0 %) |
| ***swi5∆* ^a^** | 102 | 73 (71.5 %) | 28 (27.5 %) | 1 (1.0 %) | 0 (0 %) | 0 (0 %) |
| ***sfr1∆* ^a^** | 102 | 69 (67.6 %) | 31 (30.4 %) | 2 (2.0 %) | 0 (0 %) | 0 (0 %) |
| ***rad57∆* ^a^** | 102 | 61 (59.8 %) | 40 (39.2 %) | 0 (0 %) | 1 (1.0 %) | 0 (0 %) |
| ***rqh1∆*** | 60 | 57 (95.0 %) | 1 (1.7 %) | 2 (3.3 %) | 0 (0 %) | 0 (0 %) |
| ***srs2∆*** | 90 | 78 (86.7 %) | 6 (11.1 %) | 1 (1.1 %) | 1 (1.1 %) | 0 (0 %) |
| ***fbh1∆*** | 90 | 44 (48.9 %) | 20 (22.2 %) | 0 (0 %) | 17 (18.9 %) | 9 (10.0 %) |
| ***rad51∆ fbh1∆*** | 90 | 90 (100 %) | 0 (0 %) | 0 (0 %) | 0 (0 %) | 0 (0 %) |
| ***swi5∆ fbh1∆*** | 60 | 35 (58.3 %) | 19 (31.7 %) | 1 (1.7 %) | 4 (6.7 %) | 1 (1.7 %) |
| ***sfr1∆ fbh1∆*** | 60 | 45 (75.0 %) | 8 (13.3 %) | 3 (5.0 %) | 4 (6.7 %) | 0 (0 %) |
| ***rad57∆ fbh1∆*** | 60 | 43 (71.7 %) | 15 (25.0 %) | 0 (0 %) | 2 (3.3 %) | 0 (0 %) |

For each genetic background, each assay was carried out independently at least two times, and chromosomal DNA from about 30 colonies was subjected to PFGE. Numbers in parentheses indicate the frequencies among all Ade^-^G418^s^ colonies analyzed for each strain.

^a^Data are from Akamatsu et al. (2007) [8].

GC, gene conversion; CO, crossover; BIR, break induced replication; PGFE, pulsed-field gel electrophoresis
