# Supplementary material for: Multiple Regulation of Rad51-Mediated Homologous Recombination by Fission Yeast Fbh1
Source: PLoS Genet. 2014 Aug 28;10(8):e1004542. doi: 10.1371/journal.pgen.1004542 (PMC4148199; doi:10.1371/journal.pgen.1004542)
Supplement: Table S4 — S. pombe strains used in this study. (DOC) [file pgen.1004542.s012.doc]

**Table S4**

*S. pombe* strains used in this study

| Strain | Genotype | Source/reference |
| --- | --- | --- |
| TH805 | *h* ^+^ *leu1-32 ade6-M216 ura4-D18 Ch^16^-MG* | [37] |
| TH895 | TH805 *rad51∆*::*ura4*^+^ | [37] |
| YA1056 | TH805 *swi5∆*::*ura4*^+^ | [8] |
| YA1095 | TH805 *rad57∆*::*ura4*^+^ | [8] |
| YA1316 | TH805 *sfr1∆*::*ura4^+^* | [8] |
| PS1 | TH805 *fbh1∆::his3^+^ his3-D1* | This study |
| PS2 | TH805 *srs2∆::his3^+^ his3-D1* | This study |
| PS3 | TH805 *rqh1∆::his3^+^ his3-D1* | This study |
| PS4 | TH805 *fbh1∆::his3^+^ rad51∆*::*ura4*^+^ *his3-D1* | This study |
| PS5 | TH805 *fbh1∆::his3^+^ swi5∆*::*ura4*^+^ *his3-D1* | This study |
| PS6 | TH805 *fbh1∆::his3^+^ sfr1∆*::*ura4*^+^ *his3-D1* | This study |
| PS7 | TH805 *fbh1∆::his3^+^ rad57∆*::*ura4*^+^ *his3-D1* | This study |
| TMP111 | *h^+^* *ura4-D18* *leu1-32* | [24] |
| TMP702 | *h^+^* *rhp51∆*::*ura4*^+^ *ura4-D18* *leu1-32* | This study |
| YKSPB21 | *h^+^* *fbh1∆*::*LEU2* *ura4-D18* *leu1-32* | [24] |
| C11 | *h^+^* *fbh1-fb* *ura4-D18* *leu1-32* | [27] |
| C12 | *h^+^* *fbh1-hl* *ura4-D18* *leu1-32* | [27] |
| C13 | *h^+^* *fbh1-fb/hl* *ura4-D18* *leu1-32* | [27] |
